# A pan-immune panorama of bacterial pneumonia revealed by a large-scale single-cell transcriptome atlas

**DOI:** 10.1038/s41392-024-02093-8

**Published:** 2025-01-06

**Authors:** Kun Xiao, Yan Cao, Zhihai Han, Yuxiang Zhang, Laurence Don Wai Luu, Liang Chen, Peng Yan, Wei Chen, Jiaxing Wang, Ying Liang, Xin Shi, Xiuli Wang, Fan Wang, Ye Hu, Zhengjun Wen, Yong Chen, Yuwei Yang, Haotian Yu, Lixin Xie, Yi Wang

**Affiliations:** 1 College of Pulmonary & Critical Care Medicine, The Eighth Medical Center of Chinese PLA General Hospital, Beijing, 100091 P.R. China; 2 Department of Critical Care Medicine, The Eighth Medical Center of Chinese PLA General Hospital, Beijing, 100037 P.R. China; 3 School of Life Sciences, University of Technology Sydney, Sydney, NSW 2007 Australia; 4 School of Biotechnology and Biomolecular Sciences, University of New South Wales, Sydney, NSW 2052 Australia; 5 Respiratory and Critical Care Medicine department, Beijing Jingmei Group, General Hospial, Beijing, 102308 P.R. China; 6 Department of Pulmonary and Critical Care Medicine, China Aerospace Science & Industry Corporation 731 hospital, Beijing, 100074 P.R. China; 7 Department of Respiratory Medicine, The Sixth Medical Center of Chinese People’s Liberation Army General Hospital, Beijing, 100037 P.R. China; 8 Department of Respiratory and Critical Care Medicine, Peking University Third Hospital, Beijing, 100191 P.R. China; 9 Medical School of Chinese PLA, Beijing, 100191 P.R. China; 10 Department of Pulmonary and Critical Care Medicine, Anzhen hospital afflicted to Capital medical university, Beijing, 100029 P.R. China; 11 The Eighth Medical Center of Chinese PLA General Hospital, Beijing, 100091 P.R. China; 12 Experimental Research Center, Capital Institute of Pediatrics, Beijing, 100020 P.R. China

**Keywords:** Infection, Infectious diseases

## Abstract

Bacterial pneumonia is a significant public health burden, contributing to substantial morbidity, mortality, and healthcare costs. Current therapeutic strategies beyond antibiotics and adjuvant therapies are limited, highlighting the need for a deeper understanding of the disease pathogenesis. Here, we employed single-cell RNA sequencing of 444,146 bronchoalveolar lavage fluid cells (BALFs) from a large cohort of 74 individuals, including 58 patients with mild (*n* = 22) and severe (*n* = 36) diseases as well as 16 healthy donors. Enzyme‐linked immunosorbent and histological assays were applied for validation within this cohort. The heterogeneity of immune responses in bacterial pneumonia was observed, with distinct immune cell profiles related to disease severity. Severe bacterial pneumonia was marked by an inflammatory cytokine storm resulting from systemic upregulation of *S100A8*/*A9* and *CXCL8*, primarily due to specific macrophage and neutrophil subsets. In contrast, mild bacterial pneumonia exhibits an effective humoral immune response characterized by the expansion of T follicular helper and T helper 2 cells, facilitating B cell activation and antibody production. Although both disease groups display T cell exhaustion, mild cases maintained robust cytotoxic CD8^+^T cell function, potentially reflecting a compensatory mechanism. Dysregulated neutrophil and macrophage responses contributed significantly to the pathogenesis of severe disease. Immature neutrophils promote excessive inflammation and suppress T cell activation, while a specific macrophage subset (Macro_03_M1) displaying features akin to myeloid-derived suppressor cells (M-MDSCs) suppress T cells and promote inflammation. Together, these findings highlight potential therapeutic targets for modulating immune responses and improving clinical outcomes in bacterial pneumonia.

## Introduction

Bacterial pneumonia is a significant public health concern and contributes significantly to morbidity, mortality, and healthcare costs.^1,2^ A 2021 study in China reported an age-standardized mortality rate of ~4.2 deaths per 100,000 for bacterial pneumonia, making it the fifth leading cause of death.^3^ The fatality rates are even higher in Sub-Saharan Africa, South Asia, and Southeast Asia.^3^ The incidence of severe bacterial pneumonia has increased due to factors such as antibiotic misuse and resistance, an aging population, widespread use of immunosuppressants, and the rising prevalence of chronic diseases.^4^ Approximately, one-fifth of patients hospitalized for pneumonia require intensive care unit (ICU) admission, and one-third of these patients necessitate mechanical ventilation.^5^ The mortality rates are particularly high among hospitalized patients, especially for those with severe pneumonia, reaching a mortality rate of 38%.^6^ Currently, effective treatments beyond antibiotics and adjuvant therapy are lacking for bacterial pneumonia. Therefore, a better understanding of the pathogenesis of bacterial pneumonia, especially for severe disease, is crucial for developing new therapeutic strategies.

Upon encountering pathogenic microorganisms, alveolar epithelial cells and macrophages secrete large amounts of inflammatory cytokines and chemokines, such as tumor necrosis factor (*TNF-α*), interleukin (IL)-1, *IL-6*, and CXC chemokine ligand 8 (*CXCL8*, also known as *IL-8*).^7–9^ These factors recruit and activate phagocytic cells, including neutrophils and monocytes, as well as the complement system, with the goal of eliminating pathogens. While a moderate immune response is crucial for pathogen clearance and host survival, an excessive immune response can trigger a “cytokine storm,” leading to severe pneumonia and increased mortality. Clinically, severe pneumonia is characterized by the excessive release of chemokines and cytokines, along with overexpression of adhesion molecules.^10^ This inflammatory process can damage endothelial integrity, allowing phagocytic cells, serum, and red blood cells to enter the alveolar cavity, resulting in pulmonary edema. This edema further facilitates neutrophil leakage into the alveolar cavity, promoting the release of cytokines, reactive oxygen species, and proteases.^11^ However, a systematic understanding of immune dysregulation during bacterial pneumonia remains elusive. Comprehensive and in-depth studies on the quantity, phenotype, and characteristics of various immune cells, as well as the levels of pro- and anti-inflammatory factors at different stages of disease progression, are lacking. The related disease protection or pathogenic mechanisms, especially those leading to severe disease, also require further investigation.

Understanding the alterations in the host immune system during bacterial pneumonia and elucidating the mechanisms of immune dysregulation are crucial for guiding clinical treatment, reducing the incidence and mortality of severe pneumonia, and improving patient outcomes. Single-cell RNA sequencing (scRNA-seq) has emerged as a powerful technology for dissecting the complexity of immune responses providing crucial insights into host-pathogen interactions at cellular resolution.^12–15^ Additionally, analyzing bronchoalveolar lavage fluid (BALF) from humans can provide insights into the coordinated immune response to bacterial infections like *Mycobacterium. tuberculosis*.^16^ Hence, scRNA-seq analysis of BALFs can reveal the cellular composition, transcriptional patterns, and dynamic immune responses in patients with bacterial pneumonia, shedding light on crucial inflammatory pathways and potential therapeutic targets.

**Fig. 1:**
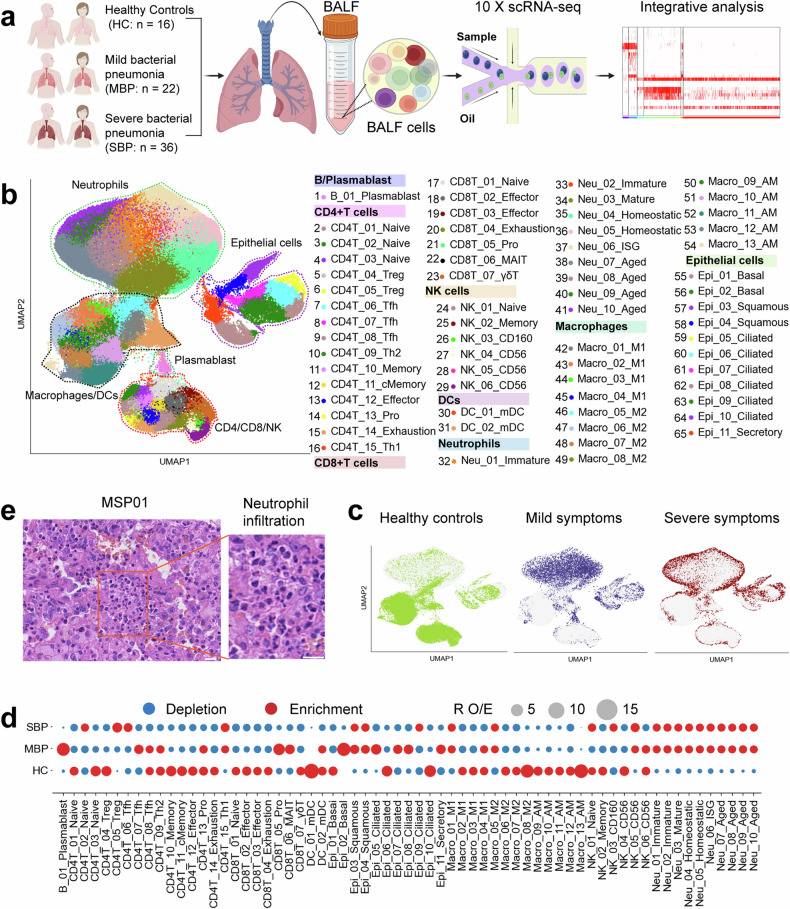
An overview of the study design and results for the BALF single-cell transcriptomic study. **a** Diagram outlining the overall study design, which included 74 individuals, including 58 patients (22 patients with mild disease and 36 patients with severe disease) and 16 healthy donors. **b** The clustering result (Left row) of 65 cell subtypes (right row) from 74 samples. Each point represents one single cell, colored according to cell type. **c** UMAP projection of the healthy donors, mild and severe. **d** Disease preference of major cell clusters as estimated using R_O/E_. **e** Representative images of hematoxylin and eosin staining of lung sections from patients with severe bacterial pneumonia (MSP01). Scale bars, 20 μm (main image; Left) and 20 μm (Magnified images, Right)

Here, we utilized scRNA-seq to obtain an unbiased visualization of the comprehensive immune response in BALF from patients with bacterial pneumonia, ranging from mild to severe symptoms, as well as from healthy controls (Fig. 1a). Our study characterizes the high-resolution transcriptomic landscape of BALF during the progression of bacterial pneumonia. Distinct immune cell profiles were observed in BALFs of individuals with bacterial pneumonia. Severe cases were characterized by heightened inflammatory responses driven by neutrophils and macrophages, while mild cases showed expansion of T follicular helper (Tfh) and T helper 2 (Th2) cells, which aided in B cell activation. The identification of cytokine and ligand-receptor interactions in these profiles has shed light on potential therapeutic targets. These findings are significant for understanding the immune mechanisms underlying different severities of bacterial pneumonia and can guide the development of novel targeted therapies to modulate immune responses and improve clinical outcomes.

## Results

### Overview of the BALF immune cell profiling in individuals with bacterial pneumonia

To characterize the immune landscape in bacterial pneumonia, single-cell transcriptomic profiles were generated from bronchoalveolar lavage fluid (BALF) samples obtained from 58 patients and 16 healthy controls (HCs) using droplet-based scRNA-seq (10x Genomics platform) (Fig. 1a). The 58 patients diagnosed with bacterial pneumonia were categorized into two clinical groups: mild bacterial pneumonia (MBP, *n* = 22) and severe bacterial pneumonia (SBP, *n* = 36). The majority of causative agents are prevalent bacterial pneumonia pathogens, such as *Acinetobacter baumannii*, *Pseudomonas aeruginosa*, and *Staphylococcus aureus*, among others. Detailed clinical information and laboratory findings of the 58 enrolled patients are provided in Supplemental Table 1 and Supplementary Fig. 1. We obtained 444,146 high-quality single cells, with an average of 3500 unique molecular identifiers (UMIs) representing 1164 genes (Supplementary Fig. 2a–d). Of these cells, 60,084 cells (13.5%) were from the HC condition, 94,590 cells (21.3%) were from the MBP condition, and 289,472 cells (65.2%) were from the SBP condition (Supplementary Fig. S2a–d). After adjusting for read depth and mitochondrial read counts, all high-quality cells were merged into an unbatched and comparable dataset for subsequent principal component analysis (See methods).

Using graph-based clustering of uniform manifold approximation and projection (UMAP), we identified transcriptomic profiles for 8 major cell types: plasmablasts, macrophages, neutrophils, CD4^+^ T, CD8^+^ T, natural killer (NK) cells, dendritic cells (DCs), and epithelial cells, based on the expression of canonical gene markers (Fig. 1b and Supplementary Fig. 2e). In the UMAP space, lymphoid cells (T and NK) were clearly differentiated from macrophages and neutrophils, whereas epithelial cells presented a distinct transcriptomic pattern from that of immune cells (Fig. 1b and Supplementary Fig. 2f). When colored by disease-level, the UMAP representation were segregated by severity following bacterial infection, particularly in severe patients compared to healthy controls, indicating bacterial infection-induced transcriptomic changes (Fig. 1c and Supplementary Fig. 3a). We firstly identified 45 cell subtypes within 8 major cell clusters (Supplementary Fig. 2f). Further subclustering revealed a total of 65 subtypes encompassing diverse respiratory cell types (Fig. 1b and Supplementary Fig. 4), which was used for subsequent analyses. Consequently, we clearly defined the cell subpopulation compositions in BALFs, providing an information-rich dataset for accurate annotation and comprehensive analysis of these cell types at different resolutions.

Each cell cluster displayed different sample origins and enrichment in disease severities (Fig. 1c and Supplementary Fig. 3). To analyze the unique immune profiles of different disease groups, we investigated the immune cell composition in each individual (Fig. 1d and Supplementary Fig. 3). Notably, the percentages of neutrophils in BALFs were significantly increased, particularly in patients with severe bacterial pneumonia (Fig. 1d, e and Supplementary Fig. 3e).^17^ In contrast to the elevation of neutrophils in BALFs, most other major immune cell types decreased in patients with bacterial pneumonia (Fig. 1d and Supplementary Fig. 3). Importantly, the preference of each cell subtype in different patient groups was also illustrated according to R_O/E_: the ratio of observed to expected cell frequencies (employed to mitigate the influence of technical variation on disease-associated cellular composition),^18^ with proliferating B (B_01_Plasmablast), CD4^+^ T (CD4T_13_Pro) and CD8^+^ T (CD8T_05_Pro) cells more enriched in mild patients compared to severe cases and healthy controls (Fig. 1d). The plasma B cells (B_01_Plasmablast) in BALFs exhibited high expression of genes encoding the constant regions of immunoglobulin G1 (IgG1), IgG3, IgG4, or IgGM (Supplementary Fig. 3f), suggesting their function in secreting antigen-specific antibodies. These findings indicate that effective B cell activation and antibody production may play an important role in bacterial pneumonia. Overall, the data revealed that each disease state in patients with bacterial pneumonia might exhibit a unique immunological fingerprint.

### Macrophage and neutrophils are the main drivers of the lung inflammatory response in patients with bacterial pneumonia

**Fig. 2:**
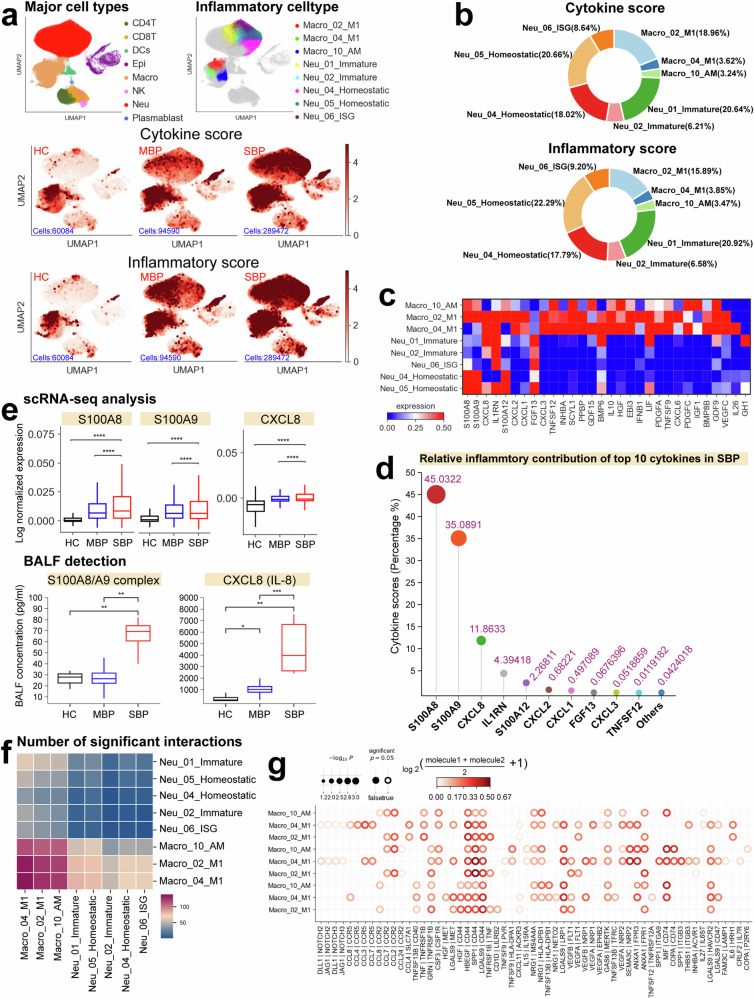
Myeloid cells are the primary contributors to the production of pro-inflammatory cytokines in severe patients. **a** UMAP projections of BALFs. Colored based on the eight major cell types (top left), eight hyper-inflammatory cell subtypes (top right), cytokine score (Middle), and inflammatory score (Bottom). **b** Pie charts depicting the relative contribution of each inflammatory cell subtype to the cytokine and inflammatory scores in severe patients. **c** Heatmap depicting the expression of cytokines within each hyper-inflammatory cell subtype identified. **d** Lollipop chart depicting the relative contribution of the top ten cytokines in patients with severe disease. **e** Box plots of cytokine expression based on scRNA-seq and plasma profiling for healthy controls, mild patients, and severe patients. Significance was evaluated using the Kruskal–Wallis test with Bonferroni correction significance was evaluated using the Kruskal–Wallis test with Bonferroni correction (*p < 0.05, **p < 0.01, ***p < 0.001, ****p < 0.0001, ^ns^*p* > 0.05). **f** Heatmap plots of the sum of significant interaction among the eight hyper-inflammatory cell subtypes. **g** Dot plot of the interactions among inflammatory macrophages in severe patients. *P* values are indicated by the circle sizes, as shown in the scale on the right

The host’s inflammatory response against the causative microorganism is crucial for resolving bacterial pneumonia.^19^ To investigate the potential origins of cytokine production, we established a cytokine score and an inflammatory score for each immune cell by analyzing the expression of cytokine genes and known inflammatory response genes, respectively (Supplementary Table 2).^20^ Cytokine and inflammatory scores were defined using the sc.tl.score_genes function in Scanpy.^12^ Hyperinflammatory cell populations were identified by performing pairwise comparisons of these scores across all subtypes using Kruskal–Wallis tests with Bonferroni correction. These two scores were utilized as indicators to evaluate the potential inflammatory impact of different immune cell populations. In patients with bacterial pneumonia, we observed significant upregulation of cytokine and inflammatory genes, indicative of an inflammatory response (Fig. 2a and Supplementary Fig. 5a). Patients with severe bacterial pneumonia exhibited significantly higher cytokine and inflammatory scores than patients with mild bacterial pneumonia, indicating the presence of an inflammatory cytokine storm in lungs of severely affected patients (Supplementary Fig. 5a).

Fourteen cell subtypes, including six subtypes of neutrophils, seven subtypes of macrophages, and one subtype of epithelial cells, exhibited significantly elevated cytokine and inflammatory scores based on our scRNA-seq data for BALFs (Supplementary Fig. 5b), suggesting their potential roles as sources of the inflammatory storm. Further analysis identified eight inflammatory cell clusters, including three macrophage clusters (Macro_02_M1, Macro_04_M1 and Macro_10_AM) and five neutrophil clusters (Neu_01_Immature, Neu_02_Immature, Neu_04_Homeostatic, Neu_05_Homeostatic, Neu_06_ISG), which exhibited significantly higher cytokine and inflammatory scores in severe patients compared to those with mild patients and healthy donors (Fig. 2a and Supplementary Fig. S6). These findings suggest that these cell clusters are likely the primary contributors to the cytokine storm observed in severe patients. We further investigated the relative abundance of these eight cell subsets in severe patients and observed a notable increase, further implicating them in the heightened inflammatory response seen in severe cases (Supplementary Fig. 5c).

Subsequent analysis revealed that four inflammatory subsets, including Macro_02_M1, Neu_01_Immature, Neu_04_Homeostatic, and Neu_05_Homeostatic, contributed over 80% of the inflammatory and cytokine scores (Fig. 2b), indicating that these cells were the predominant drivers of inflammation in severe patients. This finding aligns with previous studies on other infectious diseases such as COVID-19 where macrophages and neutrophils were also identified as key drivers of cytokine storms.^12,13^ Analysis of inflammatory signatures within the identified subsets revealed distinct pro-inflammatory cytokine gene expression profiles for each inflammatory cell cluster (Fig. 2c), including *CXCL1/2/3/8*, *S100A8/9/12*, *IL1RN*, *PPBP*, etc. Moreover, we observed elevated expression of key inflammatory cytokines, including *CXCL1/2/3* and *S100A8/9/12*, in patients with severe symptoms (Supplementary Fig. 7a). These findings suggest that diverse mechanisms may drive the inflammatory storm in severe pneumonia. The elevated expression of cell type-specific pro-inflammatory cytokines in four main subsets of inflammatory cells (Fig. 2c and Supplementary Fig. 7b) further validates their core role in driving the inflammatory storm seen in those with severe disease.

Ten pro-inflammatory cytokines (*CXCL1*/2/3/8, *S100A8/9/12*, *IL1RN*, *FGF13* and *TNFSF12*), which are primarily expressed in Macro_02_M1, Neu_01_Immature, Neu_04_Homeostatic and Neu_05_homeostatic (Supplementary Fig. 7c), are critical drivers of the inflammatory storm, accounting for over >99% of cytokine scores in severe patients (Fig. 2d). *S100A8/A9* and *CXCL8* were the most significant contributors, comprising approximately 92% of cytokine scores (Supplementary Fig. 2d). Patients with severe disease exhibited elevated expressions of *S100A8/A9* and *CXCL8* genes (Fig. 2e), further supporting our hypothesis. BALF analysis within our cohort revealed significantly higher concentrations of *S100A8/A9* complex and *CXCL8* (also known as IL-8) in severe patients, corroborating our scRNA-seq findings (Fig. 2e). This underscores the significance of the hyper-inflammatory cell subsets and molecules (*CXCL8* and *S100A8/A9*) as possible therapeutic targets for mitigating the immunopathogenesis seen in severely infected pneumonia patients.

The cytokine storm seen in severe patients may result from complex cellular crosstalk among hyperinflammatory cell subtypes, mediated by the release of various cytokines.^13^ To further investigate this phenomenon, we examined the ligand-receptor interaction patterns among eight distinct hyperinflammatory cell subsets in these patients (Fig. 2f and Supplementary Fig. 7e, f). Our analysis revealed several noteworthy ligand-receptor interactions within these subtypes, particularly highlighting the heightened interactions among inflammatory macrophages (Fig. 2f and Supplementary Fig. 7e). These inflammatory macrophages expressed multiple receptors, including *SLC7A1, CCR5, CCR2, IL15RA, HAVCR2*, etc., suggesting responsiveness to various cytokines secreted by other cells (Fig. 2g and Supplementary Fig. 7f). Notably, our analysis confirmed that the interactions between inflammatory macrophages and other hyperinflammatory cells clusters primarily rely on chemokines and their corresponding receptors (Fig. 2g and Supplementary Fig. 7f). These findings provide molecular insights into the potential mechanisms underlying complex network of interactions between hyperinflammatory cell subsets in severe pneumonia.

### Tfh and Th2 cell expansion observed in mild patients but not in severe patients

**Fig. 3:**
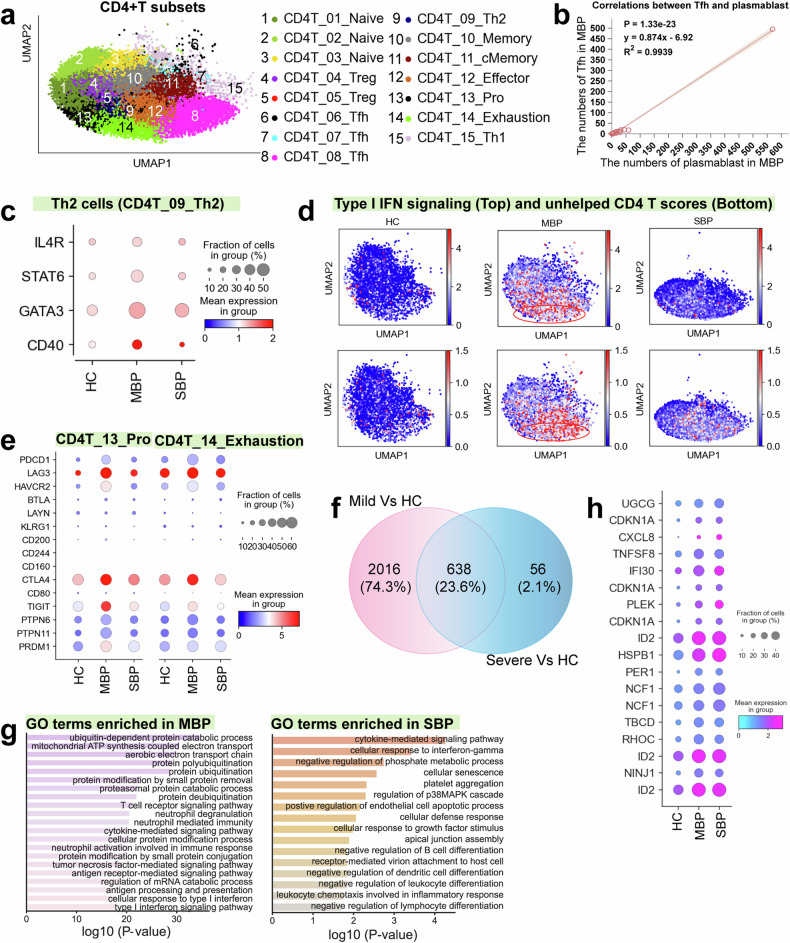
Immunological features of CD4^+^ T cell subsets. **a** The clustering result (Left row) of 15 CD4^+^ T cell types (right row) from 74 samples. Each point represents one single cell, colored according to the CD4^+^ T cell subtype. **b** Correlation between plasmablast with Tfh cells in mild patients. **c** Dot plots showing the expression of selected genes in Th2 cells across disease conditions. **d** UMAPs illustrating IFN-I response and unhelped signature scores for each CD4^+^ T cell. The red circle highlights the CD4T_14_Exhaustion cluster, which is characterized by high expression of interferon-I (IFN-I) response genes and an unhelped T cell signature. **e** Flow cytometry plots showing gating strategy and typical exhausted molecules in CD8^+^T cells from bacterial pneumonia (Top row: severe bacterial pneumonia; Bottom row: mild bacterial pneumonia). **f** Dot plots showing the cell exhaustion-related markers in CD4_13_Pro and CD4T_14_Exhaustion across disease conditions. **g** Venn diagram illustrating the number of upregulated genes in CD4^+^ T cells. **h** Enriched GO biological process terms for upregulated genes in CD4^+^ T cells from mild (Left) and severe (Right) disease. Only select terms are shown. **i** Dot plots showing CD4^+^ T cells related genes across disease conditions

A total of 15 CD4^+^ T cell subtypes were identified, including three naïve (CD4T_01_Naive, CD4T_02_Naive, and CD4T_03_Naive), two regulatory (CD4T_04_Treg and CD4T_05_Treg), three follicular helper (CD4T_06_Tfh, CD4T_07_Tfh, and CD4T_08_Tfh), Th2 (CD4T_09_Th2), memory (CD4T_10_Memory), central memory (CD4T_11_cMemory), proliferating (CD4T_13_Pro), exhausted (CD4T_14_Exhaustion) and Th1 (CD4T_15_Th1) T cells (Fig. 3a). Analysis of disease preference revealed that Tfh, Th2, and proliferating CD4^+^T cells were enriched in mild patients, while a Treg subset (CD4_05_Treg) expanded predominantly in severe patients (Fig. 1d), suggesting that a Tfh and Th2 response might be involved in controlling the bacterial infection.

CD4T_06/07/08_Tfh cells exhibited a transcriptional profile consistent with T follicular helper (Tfh) cells (e.g., high expression of inducible costimulatory; ICOS) (Supplementary Fig. 4), indicating their potential function in facilitating a protective B cell immune response. Tfh cells are crucial for generating optimal antibody responses and ensuring the production of high-quality neutralizing antibodies during pathogen infection.^21^ Consistent with this, we also observed a notable expansion of plasmablasts in individuals with mild symptoms (Fig. 1d), along with a strong positive correlation between the increased Tfh cells and expanded plasmablasts in these individuals (Fig. 3b). However, this correlation diminishes in cases of increased severity (Supplementary Fig. 8a). The primary function of Th2 cells is to facilitate B cell activation, and the cytokines they secrete enhance B cell proliferation, differentiation, and antibody production.^22^ Interestingly, among mild patients, there was a strong correlation observed between the expanded Th2 cells and the elevated plasmablasts (Supplementary Fig. 8b). Key genes and transcription factors associated with Th2 polarization and B cell activation exhibited higher expression levels in mild patients compared to both severe patients and healthy controls (Fig. 3c). Taken together, these findings suggest a coordinated response between T cells and B cells in mild patients, leading to effective humoral immunity against bacteria, which appears to become uncoupled in severe patients.

Pathogen infection can lead to lymphocyte exhaustion.^12^ We, therefore, profiled the expression of genes encoding typical markers of exhaustion in CD4^+^ T cells. Among CD4^+^T clusters, two subtypes (CD4T_13_Pro and CD4T_14_Exhaustion) exhibited notably higher exhaustion scores (Supplementary Fig. 8c), with the highest exhaustion score observed in mild patients (Supplementary Fig. 8d). The lack of substantial CD4^+^ T cell exhaustion in severe patients (Supplementary Fig. 8d) indicates that CD4^+^ T cell exhaustion may not be a significant contributor to severe bacterial pneumonia. It is well-documented that the activation of T cell exhaustion is closely connected to sustained type I interferon (IFN) signaling and inadequate CD4 + T cell assistance. In mild patients, the exhaustion profile identified within the CD4T_14_Exhaustion and CD4T_13_Pro clusters appeared to be associated with constant type I IFN signaling and insufficient assistance from CD4^+^ T cells, as anticipated (Fig. 3d). In mild cases, exhausted CD4 + T cells consistently displayed elevated expression of inhibitory genes, including *PDCD1*, *HAVCR2*, *LAG3*, *CTLA4*, and *TIGIT*, along with exhaustion-associated transcriptional factors such as *PTPN6/11* and *PRDM1* (Fig. 3e).

To identify relevant biological functions in disease-specific upregulated genes, we performed a Gene Ontology (GO) analysis of biological pathways. In CD4^+^ T cells, we detected 2654 and 694 genes upregulated for mild and severe cases versus controls, respectively, while there were 638 upregulated genes in the comparison between the two disease groups (Fig. 3f and Supplementary Table 3). The abundance of differentially expressed genes (DEGs) indicated a substantial variation within CD4^+^ T cells among mild patients. GO terms associated with upregulated DEGs in mild bacterial pneumonia patients were primarily associated with antibacterial defense and immune protection, including neutrophil activity and antigen processing, whereas those in severely affected patients were more associated with inflammatory reactions, cell apoptosis, tissue damage, and immunosuppression (Fig. 3g). Genes (e.g., *CXCL8*, *ID2*, *HSPB1*, and *PER1*), which are associated with inflammatory reactions and immunosuppression, were found to be highly expressed in severe patients (Fig. 3h). In addition, we observed that a specific subset of Treg cells (CD4T_04_Treg) exhibited high expression levels of TGF-β in patients with severe symptoms (Supplementary Fig. 8e). Treg cells are known for their role in producing TGF-β, which helps suppress CD4^+^ T cells, inhibit T cell cytokine production, and downregulates immune responses from effector cells.^23^ Consequently, the regulatory function of Treg cells appears to be associated with the development of immune tolerance and the persistence of bacterial infections in severely affected patients. Collectively, our findings suggest that the CD4^+^ T cell response in patients with mild symptoms primarily supports immune protection, whereas in severe cases, it contributes to both inflammatory reactions and immunosuppression.

### Activation of CD8^+^T cells is associated with reduced disease severity in bacterial pneumonia

**Fig. 4:**
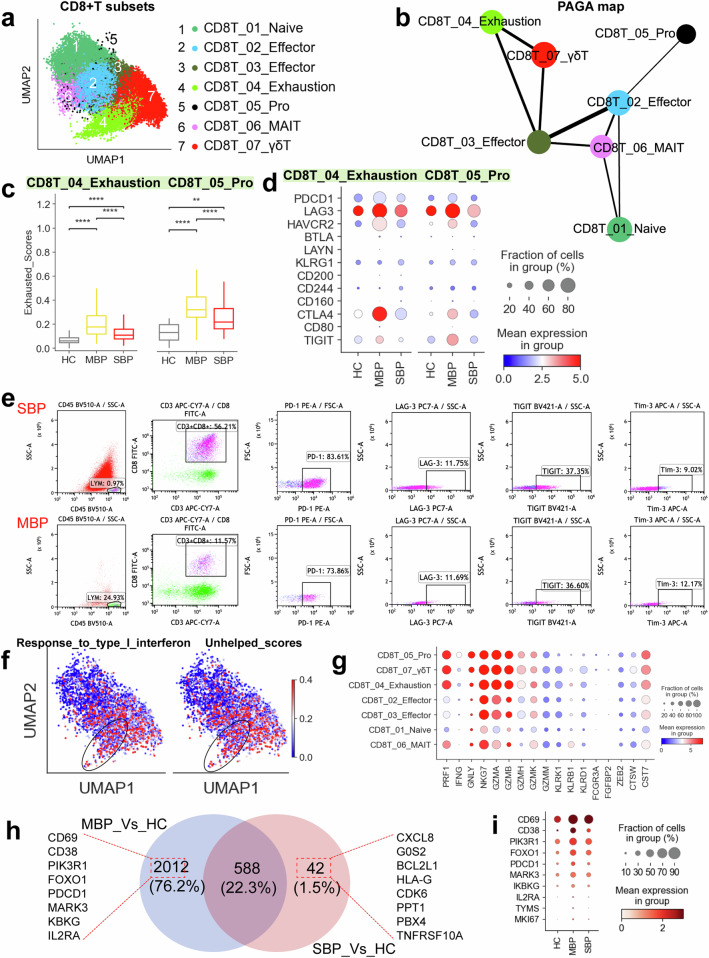
Immunological features of CD8^+^ T cell subsets. **a** The clustering result (Left row) of seven CD8^+^ T cell types (right row) from 37 samples. Each point represents one single cell, colored according to cell type. **b** PAGA analysis of CD8^+^ T cell pseudo-time: the associated cell type and the corresponding status are listed. **c** Box plots showing the exhausted scores in CD8_04_Exhaustion and CD8_05_Pro subsets across disease conditions. Significance was evaluated using the Kruskal–Wallis test with Bonferroni correction (*p < 0.05, **p < 0.01, ***p < 0.001, ****p < 0.0001, ^ns^*p* > 0.05). **d** Dot plots showing the cell exhaustion-related markers in CD8_04_Exhaustion and CD8_05_Pro subsets across disease conditions. **e** Flow cytometry plots showing gating strategy and typical exhausted molecules in CD8^+^ T cells from patients with severe (top row) and mild (below row) bacterial pneumonia. **f** UMAPs illustrating IFN-I response and unhelped signature scores for each CD8^+^ T cell. **g** Dot plots showing the cytotoxicity-related genes in CD8^+^ T cell subsets in patients with bacterial pneumonia. **h** Venn diagram illustrating the number of upregulated genes in CD8^+^ T cells. **i** Dot plots showing the activation-related genes in CD8^+^ T cell across disease conditions

Seven CD8^+^ T cell subclusters were identified in this study: naïve (CD8T_01_Naive), effector (CD8_02/03_Effector), exhausted (CD8_04_Exhaustion), MAIT (CD8_06_MAIT), γδT (CD8_07_γδT), and proliferating (CD8_05_Pro) (Fig. 4a). The CD8T_05_Pro subset, identified as highly proliferative, showed elevated levels of *MKI67* and *TYMS*, along with various effector genes (e.g., *NKG7*, *GZMA*, and *PRF1*), supporting its classification as proliferative effector CD8^+^ T cells. Analysis of disease preference indicated that mucosal-associated invariant T (MAIT) cells and proliferating CD8^+^ T cells were predominantly found in patients with mild symptoms, whereas a depletion of all CD8^+^ T cell subsets was observed in patients with severe symptoms (Fig. 1d). The reduced abundance of CD8T_05_Pro cells and its progenitor cells (CD8T_02_Effector) from individuals with severe symptoms suggests some disruption to the adaptive immune responses mediated by CD8^+^ T cells in these cases (Fig. 1d). These findings highlight important differences in CD8^+^ T cell clusters between mild and severe disease bacterial pneumonia.

A partition-based graph abstraction (PAGA) analysis was performed to examine the developmental trajectories of the seven CD8^+^ T subclusters (Fig. 4b). The analysis identified the proliferating subcluster (CD8_05_Pro) and the exhausted subcluster (CD8T_04_Exhaustion) as two distinct branches. The PAGA map revealed that two nodes (CD8T_02/03_Effector) showed high connectivity, suggesting their potential role as trans-differentiation bridges among CD8^+^ T cell subtypes. These effector states (CD8T_02/03_Effector) appear to function as intermediate stages, linking various subtypes from naive (CD8T_01_Naive) to more activated CD8^+^ T cell subclusters (e.g., CD8T_04_Exhaustion and CD8T_05_Pro). Interestingly, there was significant connectivity observed between CD8T_02_Effector and CD8T_03_Effector. The developmental trajectory towards the exhaustion and proliferation subset was also observed (Fig. 4b). This trajectory correlated with functional scores in various CD8^+^ T cell clusters, for instance, CD8T_01_Naive and CD8T_06_MAIT had high naive scores while CD8T_04_Exhaustion and CD8T_05_Pro clusters had high exhaustion scores. The highest cytotoxic score was observed in the CD8T_05_Pro cluster while the highest inflammatory score was in the CD8T_04_Exhaustion cluster (Supplementary Fig. 9).

Two subclusters (CD8T_04_Exhaustion and CD8T_05_Pro) exhibited pronounced exhaustion states (Supplementary Fig. 9), indicating that bacterial infections may lead to CD8^+^ T cell exhaustion, a critical factor contributing to the imbalance in the antibacterial infection response. Interestingly, severe patients did not display higher exhaustion scores compared to those with mild symptoms, suggesting that CD8^+^T cell exhaustion may not be a primary factor in severe bacterial infections. Consistently, CD8T_04_Exhaustion and CD8T_05_Pro cells from patients, particularly those with mild symptoms, showed increased levels of expression from various exhaustion genes (e.g., *LAG3*, *HAVCR2*, *CTLA4*, and *PDCD1*) when compared with healthy patients (Fig. 4d). *PDCD1* binds to *PD-L1/PD-L2* and *HAVCR2* (*Tim-3*) binds to galectin-9. This binding then leads to the recruitment of tyrosine-protein phosphatases *SHP1* and/or *SHP2* via their intracellular domains, including the immunoreceptor tyrosine-based switch motif (ITSM) and immunoreceptor tyrosine-based inhibitory motif (ITIM).^13^ Consequently, these interactions inhibit key signaling pathways such as PI3K-AKT and LAT-Zap70, resulting in reduced cellular proliferation and cytokine produced. Furthermore, elevated *PRDM1* levels have been associated with exhausted cells having diminished polyfunctionality and increased expression of inhibitory receptors.^13^ Correspondingly, exhausted CD8^+^ T cells from individuals with bacterial infections, especially in mild cases, had notably higher expression levels of key transcription factors such as *PRDM1* and *PTPN6/11* compared to healthy donors (Supplementary Fig. 10a). Importantly, the CD8^+^ T cell exhausted phenotypes from patients with bacterial pneumonia were confirmed with flow cytometry (Fig. 4e and Supplementary Fig. 11, 12).

The development of the exhaustion program in CD8^+^ T cells is closely associated with continuous type I interferon (IFN) signals.^24^ Our analysis demonstrated that exhausted CD8^+^ T cells had a notable enrichment of genes related to type I IFN signaling, which indicates a direct connection between the exhaustion status of CD8T_04_Exhaustion and CD8T_05 _Pro cells and persistent type I IFN signaling (Fig. 4f and Supplementary Fig. 10b). Furthermore, the exhaustion program in CD8^+^ T cells was also strongly related to compromised CD4^+^ T cell-mediated support, which helps maintain a robust CD8^+^ T cell immune response in infection.^24^ We thus analyzed whether the exhausted CD8^+^ T cells (CD8T_04_Exhaustion and CD8T_05 _Pro) exhibited signs of reduced CD4^+^ T cell help. As expected, both CD8T_04_Exhaustion and CD8T_05_Pro showed notable upregulation of transcripts typically associated with CD8^+^ T cells lacking adequate CD4^+^ T cell assistance (Fig. 4e and Supplementary Fig. 10b). Altogether, these data suggest that the exhaustion observed in the CD8T_04_Exhaustion and CD8T_05_Pro subsets may be driven by ongoing type I IFN signaling coupled with a lack of adequate CD4^+^ T cell assistance.

Despite exhibiting exhausted and “unhelped” features, exhausted CD8^+^ T cell subclusters showed obvious positive enrichment of cytotoxicity signature genes (Supplementary Fig. 9) and elevated expression levels of cytotoxic transcripts (e.g., *CST7, GNLY*, *PRF1*, *GZMA*/*B, NKG7*) (Fig. 4g), indicating that these exhausted subtypes may be heterogeneous (Supplementary Fig. 10c). These finding aligns with past reports,^24,25^ which has shown that while their capacity from proliferation and production of cytokines may be reduced, cytotoxic capabilities are often still intact in exhausted CD8^+^ T cells. Patients, especially in the mild group, displayed higher levels of cytotoxic signature gene expression in exhausted CD8^+^ T cells compared to healthy controls (Supplementary Fig. 10c). Similar to our findings in exhausted CD8^+^ T cells, we also observed notable expression of cytotoxic genes in the CD8T_07_γδT cell population (Supplementary Fig. 10c). Effector CD8^+^ T cells exhibit cytolytic functions enabling them to directly eliminate pathogens or infected cells through granule-mediated mechanisms involving perforin, granzyme, and granulysin.^12^ Therefore, the enhanced expression of various cytolytic molecules in CD8^+^ T cells may be associated with immune protection in patients with mild conditions. These findings suggest that CD8 cells have undergone complete activation, particularly in individuals with mild symptoms.

To examine the characteristics of CD8^+^ T cells in bacterial pneumonia patients further, we investigated the DEGs in individuals with mild and severe disease relative to healthy controls. In CD8^+^ T cells, we observed 2012 (76.2%) and 42 (1.5%) genes that were upregulated in mild and severe cases, respectively. (Fig. 4h and Supplementary Table S4). Additionally, a total of 588 (22.3%) genes were commonly upregulated in both disease groups (Fig. 4h and Supplementary Table S4). Notably, individuals with mild symptoms exhibited greater variability in the abundance of DEGs within CD8^+^ T cells (Fig. 4h). CD8^+^ T cells isolated from BALFs, in particular CD8T_04_Exhaustion and CD8T_05_Pro, highly expressed various activation markers (e.g., *CD69*, *CD38*, *CD25*(*IL2RA*) and *PDCD1*) in mild patients, further suggesting that CD8^+^ T cells in individuals with mild symptoms are characterized by a higher degree of activation (Fig. 4i). In contrast, DEGs in CD8^+^ T cells derived from severely ill patients exhibited elevated expression of genes linked to inflammatory response and cell apoptosis (Fig. 4i).

### Dysregulated neutrophil response as a potential contributor of severe bacterial infection

**Fig. 5:**
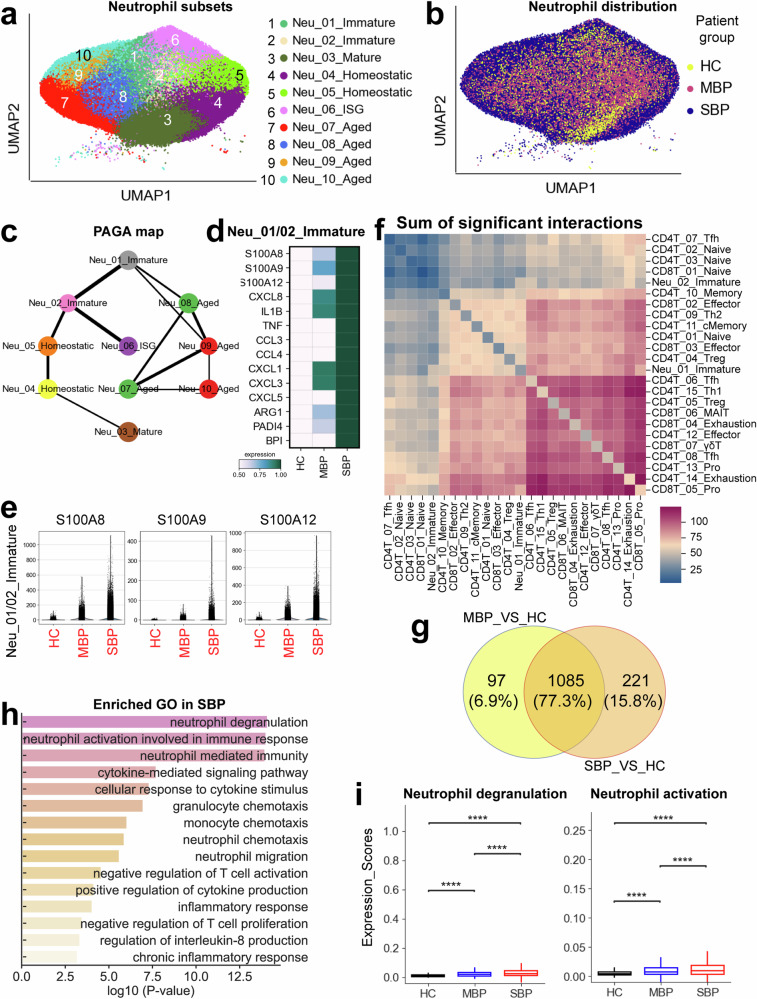
Immunological features of neutrophils. **a** The clustering result (Left row) of neutrophil subtypes (right row) from 74 samples. Each point represents one single cell, colored according to the neutrophil subtype. **b** UMAP plot of neutrophil clusters showing disease distribution. **c** PAGA analysis of neutrophil pseudo-time: the associated cell type and the corresponding status are listed. **d** Heatmap plots of selected genes in immature neutrophil subset across disease conditions. **e** Violin plots of *S100A8*, *S100A9*, and *S100A12* in immature neutrophil subset across disease conditions. **f** Heatmap plots of the sum of significant interaction between immature neutrophil clusters (Neu_01/02_Immature) and T cell subtypes in severe patients. **g** Venn diagram illustrating the number of upregulated genes in neutrophils. **h** Enriched GO biological process terms for upregulated genes in neutrophils from severe disease. Only select terms are shown. **i** Box plots showing the functional scores in neutrophils across disease conditions. Significance was evaluated using the Kruskal–Wallis test with Bonferroni correction (*p < 0.05, **p < 0.01, ***p < 0.001, ****p < 0.0001, ^ns^*p* > 0.05)

The subclustering analysis uncovered remarkable diversity within the neutrophil compartment, identifying 10 transcriptionally distinct cell subtypes. These subtypes comprised of two immature clusters, one mature cluster, four aged clusters, one interferon-stimulated genes (ISG)-related cluster, and two homeostatic clusters (Fig. 1b and Fig. 5a). All neutrophil subsets were enriched in individuals with bacterial infections (Fig. 1d, e), confirming the significant involvement of infiltrated neutrophils. This increased abundance emphasizes the crucial role of neutrophils in combatting bacterial pathogens and underscores their function as a fundamental element of the innate immune system.^26^ When visualizing neutrophils at the disease-level using UMAP, neutrophils grouped according to disease severity (Fig. 5b), suggesting that neutrophils respond differently to varying degrees of bacterial infection. PAGA trajectory analysis unveiled three distinct fates of neutrophils, with Neu_03_mature, Neu_06_ISG, and Neu_07_Aged representing distinct endpoints (Fig. 5c). The developmental trajectory was correlated with cellular maturation, interferon response, and cellular senescence (Fig. 5c), implying that neutrophils may undergo distinct developmental and functional changes after bacterial infection. These findings revealed that neutrophils exhibit varying responses depending on the severity of the bacterial infection.

Neutrophils obtained from BALF samples of patients with bacterial infections were found to contain two distinct clusters of LDNs (low-density neutrophils), designated as Neu_01_Immature and Neu_02_Immature (Fig. 5a). LDNs are primarily generated during illness (e.g., severe infection and sepsis during emergency myelopoiesis) and are strongly associated with impaired immune reactions that exhibit both immunosuppression and inflammation.^27^ Immature neutrophils derived from patients with severe bacterial infections highly expressed a range of pro-inflammatory molecules, such as *S100A8/9/12*, *CCL3/4*, *IL1B*, and *CXCL8* (Fig. 5d, e), which have been identified as key inducers of inflammatory cytokine storms in severe infectious diseases like tuberculosis and COVID-19.^13^ Immature neutrophils exhibited elevated expression of *PADI4* (Fig. 5d), a crucial factor implicated in NETs (NETosis; neutrophil extracellular trap formation). Recent studies have demonstrated the significant involvement of NETs in the pathogenesis of severe infectious diseases.^28^ In addition, immature neutrophils were found to express the *BPI* gene, which has previously been associated with adverse outcomes in severe infections.^27^ Arginase 1 (ARG1), recognized as an inhibitor of T cell activation,^27^ was also found to be highly expressed in immature neutrophils from patients with severe bacterial pneumonia (Fig. 5d). These findings indicate that immature neutrophils may contribute to the pathogenesis of severe bacterial infections in patients by promoting the formation of neutrophil extracellular traps (NETs), triggering a hyper-inflammatory response, and inhibiting the activation of T cells.

As immature neutrophils were observed to potentially hinder T cell activation, we investigated the underlying mechanisms of this suppression by examining interactions between immature neutrophils and T cells. Our analysis revealed significant interactions between immature neutrophils (Neu_01/02_Immature) and effector T cells (e.g., CD8T_05_Pro, CD8T_02/03_Effector) in patients with severe symptoms (Fig. 5f and Supplementary Fig. 13a, b). We further assessed the strength of potential ligand-receptor (L-R) pairs involved in these interactions in severely ill patients (Supplementary Fig. S13a, b). Notably, several L-R pairs, specifically HLA-E_KLRK1, HLA-E_KLRC1, HLA-E_KLRD1, and ICAM1-ITGAL, showed high interaction potentials in this patient group (Supplementary Fig. S13b). Prior evidences in chronic hepatitis B and hepatitis C virus infections have implicated HLA-E_KLRC/D/K1 signaling in driving T cell dysfunction and supporting viral persistence.^29^ Moreover, the ICAM1-ITGAL axis has been shown to inhibit T cell immune responses to infections and promote immune evasion for *Echinococcus granulosus* in mice.^30^ Collectively, these findings highlight potential targets for further investigation into the pathogenesis and therapeutic interventions for severe bacterial infections.

To further explore the functional differences in neutrophils during bacterial infections, we analyze the DEGs in neutrophils from patients with mild and severe infections (Supplementary Table S5). Our analysis revealed 1182 upregulated genes in mild cases and 1306 upregulated genes in severe cases compared to healthy controls (Fig. 5g), illustrating a clear differentiation in gene expression between mild and severe cases. Both mild and severe patient groups shared 1,085 upregulated genes. However, severe cases were characterized by an additional 221 upregulated genes, indicating a unique gene expression signature. We then focus on genes upregulated specifically in severe cases, investigating their potential role in immune damage. Overexpressed genes were enriched in pathways associated with neutrophil activation and degranulation (Fig. 5h), suggesting a heightened state of neutrophil activity in severe infections. This hyperactivation can lead to the generation of NETs, which exacerbate inflammation and tissue damage, ultimately contributing to increased disease severity (Fig. 5i). Neutrophil activation and degranulation pathways exhibited significantly elevated expression levels in severe patients (Fig. 5i). Moreover, overexpressed genes in neutrophils were also implicated in inflammatory response and T cell inhibition (Fig. 5h), further supporting the role of neutrophils in exacerbating inflammation and suppressing adaptive immunity in severe infections. Consistent with these findings, genes associated with neutrophil activation, degranulation, inflammation, and T cell inhibition were obviously upregulated in patients with severe bacterial infections (Supplementary Fig. 13c). Together, these findings reveal that neutrophil heterogeneity and their gene expression profiles may play an important role in the immune response to bacterial infections, with heightened neutrophil activity potentially linked to disease severity and dysregulation of the immune system.

### Dysregulated macrophage response as a potential contributor to severe bacterial infection

**Fig. 6:**
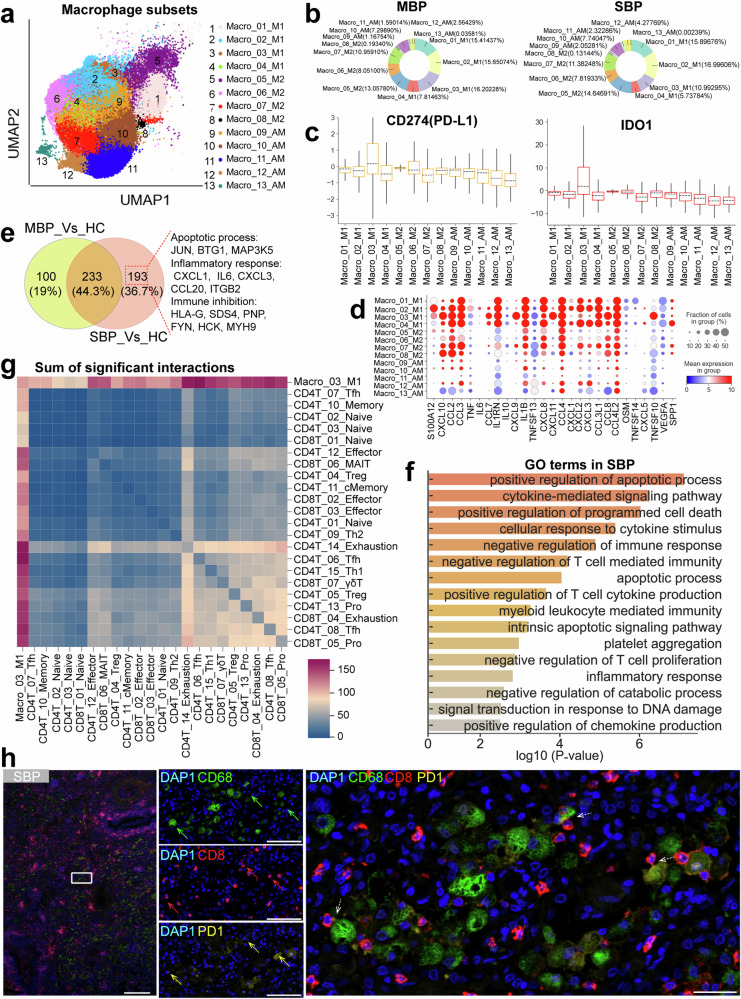
Immunological features of macrophages. **a** The clustering result (Left row) of macrophage subtypes (right row) from 74 samples. Each point represents one single cell, colored according to the macrophage subtype. **b** Pie charts depicting the relative percentage of macrophage subtype in mild (Left row) and severe (Right row) patients. **c** Box plots showing the expression scores of PD-L1 and IDO1 in macrophage subtypes. **d** Dot plots showing the inflammation-related genes in macrophage subtypes. **e** Venn diagram illustrating the number of upregulated genes in Macro_03_M1. **f** Enriched GO biological process terms for upregulated genes in Macro_03_M1 from severe disease. Only select terms are shown. **g** Heatmap plots of the sum of significant interaction between Macro_03_M1 and T cell subsets. **h** A representative multicolor immunohistochemical (IHC) stained lung section from the SBP group is shown. Macrophages, CD8^+^ T cells, and CD8^+^PD1^+^ T cells are indicated by green, red, and yellow solid arrows, respectively. Interactions between macrophages and CD8^+^ T cell subsets are highlighted with white dotted arrows. Scale bars, 2000 μm (main image; Left) and 20 μm (Magnified images, Middle and Right)

We analyzed 98,221 macrophages from three distinct disease conditions, revealing significant remodeling of macrophage populations in patients with bacterial infections compared to healthy controls (Fig. 6a and Supplementary Fig. 14a, b). To explore their heterogeneity further, we re-clustered 13 macrophage subtypes (Figs. 1b, 6a). Based on the most recent classifications and frequently used markers,^31,32^ macrophages were categorized according to their expression patterns of *MARCO*, *FABP4, MRC1*, *SPP1*, and *FCN1* (Supplementary Figs. 2, 4). These categories included the classical monocyte-like macrophage group (Macro_01/02/03/04_M1), the M2-like macrophage group (Macro_05/06/07/08_M2), and the alveolar-like macrophage group (Macro_09/10/11/12/13_AM). In healthy individuals, the AM-like population comprised over 70% of lung macrophages (Supplementary Fig. 14c), while in patients with bacterial pneumonia, M1- and M2-like macrophages were predominant and accounted for approximately 80% of total lung macrophages (Fig. 6b). There was also a significant reduction in various AM-like subsets in patients with bacterial pneumonia, indicating a significant shift in macrophage composition after infection (Supplementary Fig. 14b).

Given the substantial remodeling towards M1- and M2-like macrophages in patients with bacterial infections (Fig. 6b and Supplementary Fig. 14a–c), we analyzed these cellular compartments in greater detail. A macrophage cluster, identified as Macro_03_M1, exhibited elevated levels of *IDO1* and *PD-L1* (*CD274*) (Fig. 6c). Increased *PD-L1* and *IDO1* levels are commonly linked to immunosuppressive activities of macrophagic myeloid-derived suppressor cells (MDSCs), neutrophils, granulocytes, and monocytes.^27^ Moreover in lung tuberculosis, IDO1^+^ macrophages have been associated with suppressive functions.^16^ This suggests that the Macro_03_M1 subcluster in bacterial infections resembled macrophagic MDSCs (M-MDSCs), highlighting its potential role in immune suppression and the maintenance of bacterial infections, potentially contributing to disease progression. Beyond its role in immune suppression, M-MDSCs (Macro_03_M1) also produce various pro-inflammatory cytokines, which can exacerbate tissue damage. Macro_03_M1 and other M1- and M2-like macrophage clusters exhibited high expression of various pro-inflammatory cytokines (Fig. 6d). We identified substantial expression of classical pro-inflammatory cytokine genes like *S100A12*, *TNF*, *IL1B*, *IL6*, *CXCL8*, *CXCL1*, and *CXCL3* by M1- and M2-like macrophages in severely ill patients (Supplementary Fig. 14d). By defining a pro-inflammatory cytokine score based on these upregulated cytokines (Fig. 6d), we observed that most M1-like and M2-like macrophage subsets in severe bacterial infections exhibited notably elevated pro-inflammatory cytokine scores (Supplementary Fig. 14e), implying that they are important drivers of the inflammatory response which may lead to increased tissue damage and disease severity. Collective, these findings highlight that M1- and M2-like macrophages may play pivotal roles in both immune suppression and inflammation, contributing to the difficulty in managing bacterial infections and leading to the worse prognosis observed in severely ill patients.

To better understand the functional differences in the Macro_03_M1 cluster between bacterial-infected patients and healthy controls, we conducted DEG and GO analysis. Within the Macro_03_M1 cluster, we identified 333 genes upregulated in patients with mild symptoms and 426 genes upregulated in those with severe symptoms (Fig. 6e and Supplementary Table S6), illustrating a significant escalation in gene activity as the disease progresses. The two patient groups shared 233 upregulated genes (Fig. 6e). Notably, severe cases were characterized by an additional 193 upregulated genes (Fig. 6e). GO analysis revealed significant enrichment in genes associated with immune inhibition such as ‘negative regulation of immune response’, ‘negative regulation of T cell activation/proliferation’ and ‘negative regulation of T cell-mediated immunity’, in severe patients. This further supports the concept that M-MDSCs (Macro_03_M1) represent a diverse myeloid cell subset with robust immunosuppressive capabilities.^12^ M-MDSCs are capable of directly suppressing the T cell activation and function,^13^ which are essential components of the adaptive immune system. Therefore, this suppression by the Macro_03_M1 cluster may contribute to a reduced immune response against bacterial infections in severe patients. Consistent with these data, the Macro_03_M1 cluster exhibited elevated expression of multiple genes associated with immunosuppressive functions (e.g., *RIPOR1*, *SDC4*, *FGR*, and *PNE*) (Supplementary Fig. 15a). In severe patients, Macro_03_M1 also displayed activated inflammation and apoptosis phenotypes, with high levels of various genes related to inflammation and apoptosis (e.g., *IL6*, *CXCL1*, *CXCL3*, *JUN*, and *MAP3K5*) (Supplementary Fig. 15b). Hence, the Macro_03_M1 cluster in severe patients is marked by a combination of immunosuppression, inflammation and apoptosis, highlighting its multifaceted role in the pathology of severe infections.

Next, we examined the intercellular interactions between T cells and Macro_03_M1. Our data revealed that the Macro_03_M1 cluster predominantly interacted with exhausted and effector T cells in severe patients (Fig. 6g and Supplementary Fig. 15c). We further confirmed the adjacent spatial relationship between macrophages and exhausted T cells in patients with severe bacterial pneumonia by multicolor immunohistochemistry (IHC) staining (Fig. 6h and Supplementary Fig. 16). We next assessed the strength of potential ligand-receptor (L-R) pairs involved in the interactions between Macro_03_M1 and both exhausted and effector T cells in severe patients (Supplementary Fig. 15d). Multiple ligand-receptor pairs between killer immunoglobulin-like receptor family (KIR; e.g., KIR3DL1/2) and HLA class I (HLA-I; e.g., HLA-A/F/B) exhibited high interaction potentials. Recent studies have demonstrated that the HLA-I_KIR axis is pivotal in mediating T cell dysfunction and inhibiting T cell activation, cytokine production, and cytotoxicity.^33,34^ In addition, several chemokines (e.g., *CCL2/3/4/7/8*) and their receptors (e.g., *CCR1/2/5/8*) also displayed strong potential interaction (Supplementary Fig. 15d). The interactions between chemokines and their receptors (e.g., CCL3_CCR5 axis) is crucial for directing T cells to the sites of infection.^35^ During infection, persistent signaling through CCR5 can lead to continuous T cell activation, potentially resulting in T cell exhaustion and reduced functionality.^36^ Thus, understanding these interactions between M-MDSCs (Macro_03_M1) and T cell subtypes provides insights into potential therapeutic strategies to enhance immune function in severe bacterial pneumonia.

## Discussion

In this study, scRNA-seq was employed to generate a comprehensive picture of the lung microenvironment immune landscape with single-cell resolution during bacterial pneumonia. A large cohort of 74 samples (Fig. 1), including 22 with mild pneumonia, 36 with severe pneumonia, and 16 healthy control samples, was analyzed, revealing distinct immune cell profiles associated with disease severity. These findings provide greater understanding of the pathogenesis in bacterial lung infections across different disease severities and assist in identifying new immunological targets for the treatment of bacterial pneumonia. While this study focused on bacterial pneumonia, comparing our findings with datasets from viral pneumonia, such as COVID-19,^20^ revealed distinct immune signatures characteristic of bacterial infection. These signatures, including neutrophil activation and macrophage polarization patterns (Figs. 5, 6), could inform the development of improved diagnostic and therapeutic strategies.

Our analysis first identified eight major cell types, with a total of 65 cell subtypes, providing a detailed landscape of the respiratory immune system in bacterial pneumonia (Fig. 1). Notably, a significant increase in neutrophil abundance was observed, particularly in patients with severe bacterial pneumonia, highlighting their role in the pathogenesis of the severe disease. Consistent with previous reports, our study confirms that neutrophil accumulation, while critical for host defense against bacterial infection, is a pathological hallmark of bacterial pneumonia and a contributing factor to lung injury.^37–39^ Importantly, we observed a significant difference in the degree of pulmonary neutrophilia between patients with severe and mild bacterial pneumonia, with more pronounced neutrophil infiltration in the severe cases (Fig. 1). In contrast, a decrease in the proportion of most other major immune cell types was found in patients, particularly in those with severe bacterial pneumonia, implying a potential suppression of the adaptive immune response. Further analysis revealed that CD4^+^ T, CD8^+^ T, and proliferating B cells were uniquely enriched in mild patients, suggesting a more robust adaptive immune response in these individuals. This is supported by plasma B cells showing greater expression of genes that encode the IgG constant regions, indicating their role in secreting antigen-specific antibodies. Collectively, these results highlight the heterogeneity of immune responses in bacterial pneumonia, with distinct immune cell profiles related to disease severity.

Our study provides a detailed overview of the pulmonary inflammatory landscape in patients with bacterial pneumonia, revealing a specific macrophage- and neutrophil-driven cytokine storm in severely ill patients (Fig. 2). This is the first study to provide evidence of a cytokine storm within the lungs of patients with severe bacterial pneumonia, characterized by a comprehensive analysis of the specific cell populations and key inflammatory cytokines involved. While a general increase in cytokine and inflammatory markers was observed in patients with bacterial pneumonia,^40^ our scRNA-seq analysis of BALFs provided detailed insights into the specific cell populations contributing to the heightened inflammatory response. Eight hyperinflammatory cell subsets were identified in patient BALF, indicating targeted rather than global inflammatory dysregulation. Notably, four of these subsets, including one macrophage subset (Macro_02_M1) and three neutrophil subsets (Neu_01_Immature, Neu_04_Homeostatic, and Neu_05_Homeostatic), were identified as the primary contributors to the inflammatory cytokine storm. These subsets collectively accounted for over 80% of the inflammatory signature and exhibited a marked increase in abundance within the severe patient cohort. Intriguingly, despite their shared role in driving the cytokine storm, these subtypes displayed distinct pro-inflammatory cytokine profiles, highlighting the diverse mechanisms contributing to disease severity. Specifically, *S100A8/A9* and *CXCL8*, primarily produced by inflammatory neutrophil subtypes, were central mediators of the cytokine storm, a finding corroborated by both gene expression and plasma protein level validation. Importantly, extensive chemokine-mediated crosstalk was observed between these hyperinflammatory subtypes, particularly involving inflammatory macrophages expressing a diverse repertoire of chemokine receptors. This complex interplay likely amplifies the inflammatory response, exacerbating lung damage. Hence, targeted therapeutic strategies aimed at these specific inflammatory subsets, potentially through modulation of *S100A8/A9* and *CXCL8*, or their associated signaling pathways, may offer more effective treatment strategies for mitigating the severe inflammatory pathology associated with bacterial pneumonia. Further studies are warranted to investigate these targeted therapies and elucidate the detailed mechanisms underlying this intricate inflammatory crosstalk.

For T cells, our results revealed a dichotomous role of their responses in bacterial pneumonia, with mild cases characterized by robust Tfh, Th2, and cytotoxic CD8^+^T cell responses that likely contribute to effective bacterial clearance, while severe cases exhibit features of immune dysregulation and impaired T cell function. The expansion of Tfh cells, in conjunction with increased plasmablasts and upregulation of genes related to B cell activation, suggests an effective humoral immune response in mild patients. In particular, this coordinated Tfh-B cell axis, which is crucial for producing high-affinity antibodies and establishing long-term immunity, appears disrupted in severe cases, potentially contributing to disease progression. While both mild and severe cases exhibited T cell exhaustion, characterized by upregulated inhibitory receptors (e.g., *PD-1*, *LAG3*, *CTLA4*, and *TIM-3*) and exhaustion-associated transcription factors (*PTPN6/11*, *PRDM1*), the functional consequences differed. In mild cases, exhausted CD8^+^ T cells retained robust cytotoxic function, evidenced by high expression of cytotoxic molecules (e.g., *PRF1*, *GZMA/B*, and granulysin), potentially reflecting a compensatory mechanism to maintain control over bacterial replication. In contrast, severe cases showed diminished CD8^+^ T cell responses, with fewer proliferating and effector CD8 + T cells, suggesting impaired adaptive immunity. This impaired response, coupled with the expansion of a specific Treg subset (CD4T_04_Treg) expressing high levels of immunosuppressive *TGF-β*, further points towards an immunosuppressive milieu in severe cases that likely hinders bacterial clearance. Therefore, our results highlight the importance of dissecting disease heterogeneity of T cells in bacterial pneumonia, as targeting specific T cell subsets or modulating the T cell response may offer tailored therapeutic strategies for different disease severities.

scRNA-seq analysis reveals a complicated and dysregulated neutrophil response in bacterial pneumonia, with distinct neutrophil subtypes and gene expression patterns linked to the severity of the disease. Although neutrophil infiltration is a common occurrence in bacterial infections,^41^ immature neutrophils, particularly LDNs, play a role in exacerbating an overly inflammatory condition in severe cases. These immature neutrophils exhibit heightened levels of typical pro-inflammatory substances like *S100A8/9/12* and *CXCL8*, along with markers of NETosis such as *PADI4*, which likely fuel the cytokine storm seen in severe infections. Furthermore, the increased expression of *ARG1* in immature neutrophils and their interaction with effector T cells via *HLA-E-KLRC/D/K1* and *ICAM1/ITGAL* axes suggest a potential involvement in suppressing T cell responses, further compromising adaptive immunity in severe patients. The dysregulated neutrophil response, marked by excessive inflammation and compromised T cell activity, highlights a potential mechanism driving disease severity in bacterial pneumonia. Collectively, these data emphasize the importance of targeting specific neutrophil subtypes and inflammatory pathways as a potential therapeutic strategy for severe cases of bacterial pneumonia.

Our analysis underscores the dual role of macrophages in bacterial pneumonia, contributing to both immune dysregulation and inflammation, particularly in those with severe manifestations. In infected lungs, alveolar macrophages were found to be depleted while M1- and M2-like macrophages increased. Notably, the Macro_03_M1 cluster, showing features akin to M-MDSCs, is of significant interest. These cells displayed elevated expression of immunosuppressive molecules (*PD-L1* and *IDO1*), suggesting their involvement in suppressing T cell responses, a characteristic feature of severe disease. This immunosuppressive function was further corroborated by their interaction with exhausted and effector T cells via the HLA-I-KIR axis, which is known to inhibit T cell activation and function. Additionally, these M-MDSCs also had a pro-inflammatory profile, with elevated pro-inflammatory molecules (including *S100A12*, *TNF*, *IL1B*, *IL6*, and *CXCL8*) that likely contributed to the exacerbation of lung inflammation and tissue damage. This dual functionality of M-MDSCs, simultaneously suppressing T cells and promoting inflammation, represents a critical mechanism underlying the severity of bacterial pneumonia. Hence, targeting these dysregulated macrophages, either by inhibiting their immunosuppressive function or mitigating their inflammatory capacity, may offer a promising therapeutic avenue for severe cases of bacterial pneumonia.

There are important limitations to the interpretation of our study. First, this study used BALF samples taken from only one timepoint throughout the patients’ illness, thereby offering a limited perspective on its dynamic progression. Hence, further investigations incorporating longitudinal sampling are recommended to capture the temporal variations in immune cell populations and gene expression profiles throughout the infection and recovery phases. Second, our study focused on the analysis of BALF, which primarily reflects the immune environment of the alveolar region. Nevertheless, bacterial pneumonia may also affect other lung compartments (e.g., interstitium). Herein, incorporating analysis of additional lung regions may provide a more comprehensive insight into the immune response landscape in bacterial pneumonia.

Taken together, our study comprehensively maps the immune landscape of the lung microenvironment in bacterial pneumonia, revealing distinct immune profiles correlated with disease severity. Severe bacterial pneumonia was characterized by a heightened inflammatory response primarily driven by specific macrophage and neutrophil subsets. Conversely, mild bacterial pneumonia exhibited a robust Tfh and Th2 cell response that promotes effective humoral immunity. Dysregulated neutrophil and macrophage responses contribute significantly to the pathogenesis of severe disease. These findings highlight potential therapeutic targets for modulating immune responses and improving clinical outcomes in bacterial pneumonia.

## Materials and methods

### Ethical approval

This study was conducted in accordance with the Declaration of Helsinki and received ethical approval from the Ethics Committee of the General Hospital of the People’s Liberation Army of China (Ethics No. 309202305011312). All patients or their legal representatives provided written informed consent prior to participation.

### Study design and participants

This study included 58 patients diagnosed with bacterial pneumonia and 16 healthy controls (Supplementary Table 1), where 6 of the healthy controls were recruited in this study, while the remaining 10 were sourced from a prior study.^42^ Participants were recruited from the intensive care units of seven hospitals in Beijing between October 2023 and March 2024. Diagnosis of bacterial infections was confirmed through polymerase chain reaction (PCR) testing, high-throughput pathogen sequencing, or culture-based assays. Pneumonia diagnoses were based on the presence of characteristic clinical features, including cough, fever, sputum production, and pleuritic chest pain, supported by chest radiography.^43^ While physical examination findings, such as rales or bronchial breath sounds, contribute to the clinical evaluation, they are considered less sensitive and specific than radiographic imaging for confirming pneumonia.^44^

Severe pneumonia was defined according to the 2007 Infectious Diseases Society of America/American Thoracic Society (IDSA/ATS) criteria for severe community-acquired pneumonia.^43^ This validated definition requires the presence of either one major criterion or three or more minor criteria, as outlined below: (1) Major Criteria: Septic shock requiring vasopressor support; Respiratory failure necessitating mechanical ventilation; (2) Minor Criteria: Respiratory rate >30 breaths/min; PaO2/FiO2 ratio <250; Multilobar infiltrates; Confusion/disorientation; Uremia (blood urea nitrogen level >20 mg/dL); Leukopenia (white blood cell count <4000 cells/mL); Thrombocytopenia (platelet count <100,000/mL); Hypothermia (core temperature <36.8 °C); Hypotension requiring aggressive fluid resuscitation.

Patients were excluded if they met any of the following criteria: (1) Age <18 years; (2) Viral pneumonia; (3) Autoimmune diseases; (4) Immunosuppression (e.g., receiving corticosteroids or chemotherapy, undergoing transplantation, hematologic malignancies, HIV infection with CD4^+^ T count <200 cells/mL); (5) Comorbidities (e.g., asthma, chronic obstructive pulmonary diseases, pulmonary cystic fibrosis, and bronchiectasis).

Bronchoalveolar lavage fluid (BALF) samples were collected immediately after patients were diagnosed with mild or severe bacterial pneumonia. To minimize the impact of regional heterogeneity, BALF was performed on the lung segment exhibiting the most severe or recent radiographic evidence of infection, as determined by CT or chest X-ray. This targeted sampling strategy aimed to ensure that the collected BALF reflected the most clinically relevant aspects of the patient’s pulmonary infection.

### Single-cell RNA sequencing and data analysis

BALF samples from patients diagnosed with bacterial pneumonia underwent immediate processing (within 2 h). To generate single-cell suspensions, BALF was filtered using a 100-μm nylon cell strainer and subsequently suspended in a chilled, complete RPMI 1640 medium. These freshly isolated cells then underwent 10x Genomics single-cell RNA sequencing. To mitigate the risk of blood cell contamination, red blood cells were lysed using a dedicated lysis buffer during the BALF processing procedure. Furthermore, the expression of blood cell markers, such as HBB, was rigorously assessed during the data quality control stage to ensure the absence of contamination within the final BALF dataset. Single-cell RNA sequencing libraries were prepared using the Chromium Single Cell 5’ Kit v2 (10x Genomics; PN-1000263) following the manufacturer’s protocol. Sequencing was performed on an Illumina NovaSeq 6000 sequencer with 2x150bp paired-end reads.

Single-cell RNA sequencing (scRNA-seq) data analysis was conducted as described.^12,13^ Briefly, a python wrapper (kb v0.24.4) for kallisto and bustools and the anndata ad.concat function (v0.7.6) was used to generate a merged and filtered gene expression matrix which encompassed all samples.^12^ Scanpy (sc) (v1.9.2) was then to (1) remove low-quality and doublet cells, (2) normalize library size to 10,000 reads per cell, and (3) identify the top most highly variable genes (HVGs) expressed between cells.^12^ To enable unsupervised clustering of cells from different samples in a shared transcriptional space, batch effect correction was performed using the Harmony algorithm.^45^ Prior to Harmony integration, variable gene selection was performed independently for each sample. A consensus list of 1500 highly variable genes was then generated by selecting genes with the highest recovery rates across all samples, resolving ties through random selection. Ribosomal, mitochondrial, and immunoglobulin genes were excluded from this final list of variable genes. Next, a principal component analysis (PCA) matrix with 20 components was calculated using the selected informative genes. This PCA matrix was then used as input for batch effect correction with Harmony, implemented using the external.pp.harmony_integrate function in Scanpy.^45,46^ Sample and dataset variables were set as technical covariates for correction with theta values of 2.5 and 1.5, respectively. The resulting batch-corrected matrix was used to construct a nearest neighbor graph in Scanpy, which was subsequently used for cell clustering via the Louvain algorithm.^47^ Cluster-specific marker genes were identified using the rank_genes_groups function .

### Cell clustering and annotations

Unsupervised cell clustering was performed in two consecutive rounds with the function: sc.tl.louvain. The first round (Louvain resolution 2.0) successfully delineated eight major cell types: plasmablasts, macrophages, neutrophils, CD8^+^ T, CD4^+^ T, natural killer (NK), dendritic cells (DCs), and epithelial cells. Canonical marker genes were then used to manually annotate subclusters within these major cell types. These subclusters are representative of distinct immune cell lineages. Next, the sc.tl.rank_genes_groups function was used to identify cluster-specific signature genes, which were then cross-referenced to established gene markers to confirm correct annotations.

Cellular trajectories were inferred using partition-based graph abstraction (PAGA) implemented in Scanpy (v1.5.1) with default parameters.^48^ PAGA analysis revealed a continuous transition between discrete cell types. Differentially expressed genes (DEGs) were identified using the sc.tl.rank_genes_groups function in Scanpy with the parameter use_raw=True. DEGs were determined based on cluster membership or disease condition using the Wilcoxon rank-sum test with an adjusted *p* value <0.01 and a fold-change threshold >1.5. CellPhoneDB analysis of ligand-receptor interactions was performed with parameters set to alpha = 0.01 and *p* value threshold = 0.01.

### Identifying changes in immune cell proportion

The Kruskal–Wallis test with Bonferroni correction was used to calculate the statistically significant differences in the relative abundance of immune cell types and subtypes across disease conditions. Furthermore, a multivariate ANOVA was employed to assess the influence of disease stage on the proportion of different cell types and subtypes.^13^ To elucidate associations of specific cell populations with particular disease phases, a ratio of observed to randomly expected cell numbers (R_O/E_) was calculated, providing a measure of disease preference.^13^

### Determining cell state scores

The overall physiological activities and activation level for each cell type and subtype in each disease phase were compared using a gene set scoring approach. Gene sets representing pro-inflammatory cytokines, inflammatory responses, naïve states, exhaustion states, cytotoxic activity, regulatory effector functions were compiled from previously published studies (Supplementary Table S2).^12,49,50^ Cell state scores were computed using the sc.tl.score_genes function in Scanpy, which normalizes the expression of genes within predefined gene sets to reference genes. Statistical comparisons of cell state scores between disease phases were performed using the Kruskal–Wallis test with Bonferroni correction.

### Plasma cytokine quantification and flow cytometry

Levels of cytokines in plasma were determined with the Th1/Th2 34-plex human ProcartaPlex kit (Thermo Fisher Scientific) as described previously.^51^ Flow cytometry were also performed following the manufacturer’s instructions as described previously.^50^

### Histological examination

Central and peripheral lung tissue sections from each lobe were fixed in 4% formaldehyde, embedded in paraffin (FFPE), and processed for histological examination. Sections (1 mm thick) were cut from FFPE blocks using a Tissue-Tek Prisma Plus automated staining system and Tissue-Tek Film (Sakura) following standard protocols. Sections were deparaffinized, rehydrated, and stained with hematoxylin and eosin (H&E).

### Multicolor Immunohistochemistry

Freshly harvested tissues were fixed in 4% neutral buffered formalin and embedded in paraffin. Multiplex immunofluorescence staining was performed on 4-μm tissue sections using the PANO 7-plex IHC Kit (cat#0004100100, Panovue) according to the manufacturer’s protocol. Briefly, sections were deparaffinized, rehydrated, and subjected to heat-mediated antigen retrieval in citrate acid buffer (pH 6.0) using microwave incubation. Following a 10-min incubation with a blocking buffer at room temperature, sections were incubated with primary antibodies against CD8 (CST70306, CST), PD-1 (CST43248, CST), and CD68 (CST76437, CST). Sections were then incubated with a horseradish peroxidase-conjugated secondary antibody (anti-rabbit or anti-mouse) for 10 min at room temperature. Signal amplification was performed using tyramide signal amplification reagents (0004100100, Panovue). Nuclei were counterstained with 4’,6-diamidino-2-phenylindole (DAPI), and stained slides were scanned using the Mantra System (PerkinElmer).

### Statistical analysis

R and Python were used for statistical analyses and visualization. For all figures, the following symbols were used to represent *p* values: ns (*p* > 0.05), *p ≤ 0.05, **p ≤ 0.01, ***p ≤ 0.001, ****p ≤ 0.0001.

## Supplementary information

Supplementary Information 01

Supplementary Information 02

## Data Availability

The data that support the findings of this study are openly available in the China National Center for Bioinformation at https://ngdc.cncb.ac.cn/omix/release/OMIX007986; reference number OMIX007986. The data reported in this paper have been deposited in the OMIX, China National Center for Bioinformation/Beijing Institute of Genomics, Chinese Academy of Sciences (https://ngdc.cncb.ac.cn/omix; accession no. OMIX007986).

## Code availability

Customized data analysis scripts utilized in this study are available upon reasonable request.
