# Supplementary material for: A pan-immune panorama of bacterial pneumonia revealed by a large-scale single-cell transcriptome atlas
Source: Signal Transduct Target Ther. 2025 Jan 6;10:5. doi: 10.1038/s41392-024-02093-8 (PMC11701081; doi:10.1038/s41392-024-02093-8)
Supplement: Supplementary file 1 — Supplementary Information 01 [file 41392_2024_2093_MOESM1_ESM.docx]

**Supplementary Materials for**

**A pan-immune panorama of bacterial pneumonia revealed by a large-scale single-cell transcriptome atlas**

Kun Xiao ^1,^***^,🖂^**, Yan Cao ^1,^*, Zhihai Han ^1,^*, Yuxiang Zhang ^2,^*, Laurence Don Wai Luu ^3,^*, Liang Chen ^4^, Peng Yan ^5^, Wei Chen ^1,6^, Jiaxing Wang ^2^, Ying Liang ^7^, Xin Shi ^1,8^, Xiuli Wang ^1,8^, Fan Wang ^1^, Ye Hu ^1^, Zhengjun Wen ^4^, Yong Chen ^9^, Yuwei Yang ^1^, Haotian Yu ^10,^**^🖂^**, Lixin Xie ^1,^**^🖂^**, Yi Wang ^11,^**^🖂^**

Correspondence to: [13716608331@163.com](mailto:13716608331@163.com); [yht200725@163.com](mailto:yht200725@163.com); [xielx301@126.com](mailto:xielx301@126.com); [wildwolf0101@163.com](mailto:wildwolf0101@163.com).

**The file includes:**

**Supplementary Figure 1-16**

**
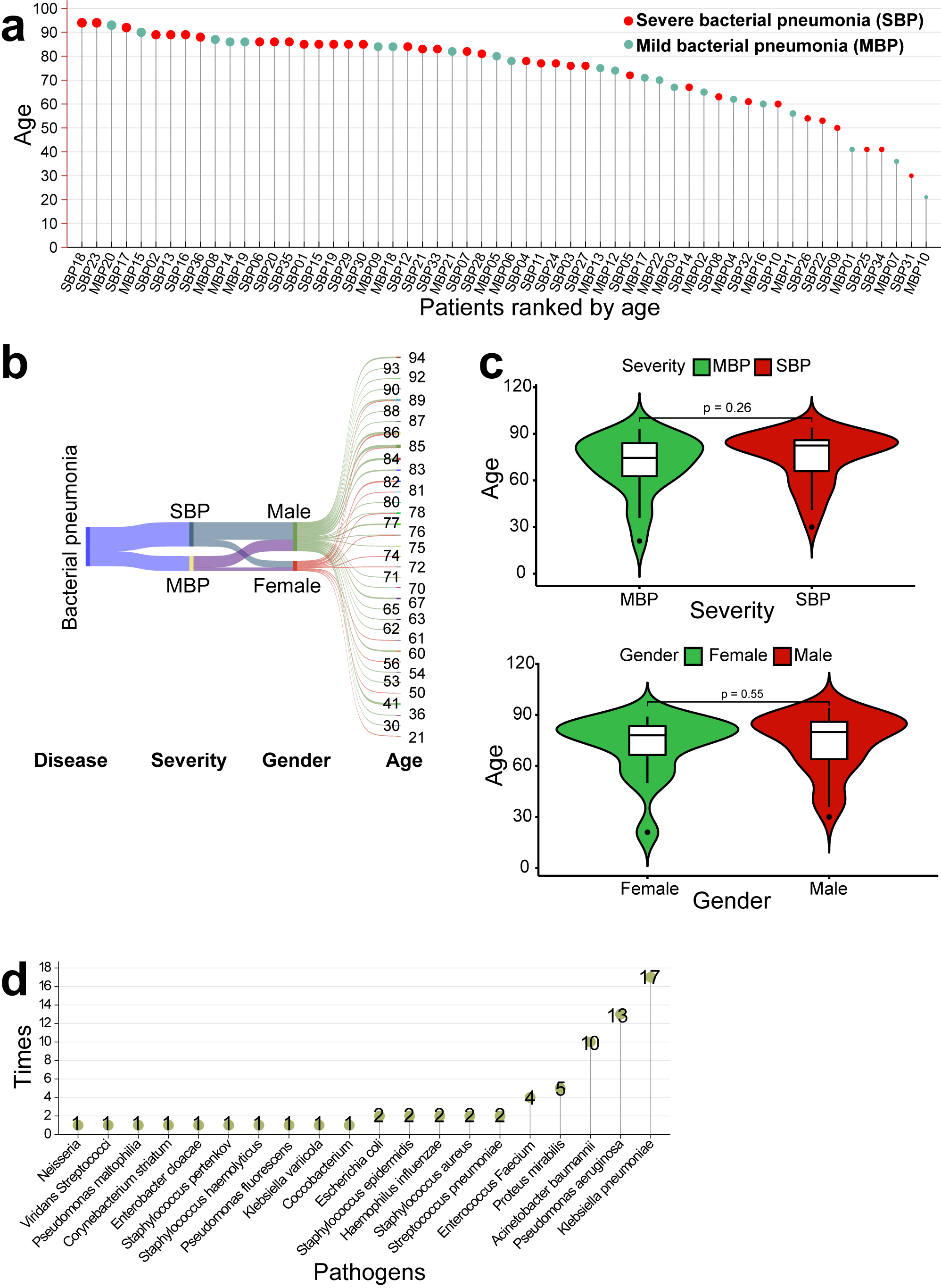
**

**Supplementary Fig 1. Clinical characteristics and pathogen distribution of the integrated dataset.**

a. The age distribution of the dataset (color-coded by disease conditions).

b. Sankey diagram showing information on the distribution of bacterial pneumonia cases according to severity, gender, and age.

c. Box plots depict the age distributions of bacterial pneumonia patients stratified by disease severity (top) and gender (bottom). No statistically significant differences in age were observed between patients with mild and severe bacterial pneumonia (p = 0.26) or between male and female patients (p = 0.55).

d. The pathogen distribution of the dataset.

**
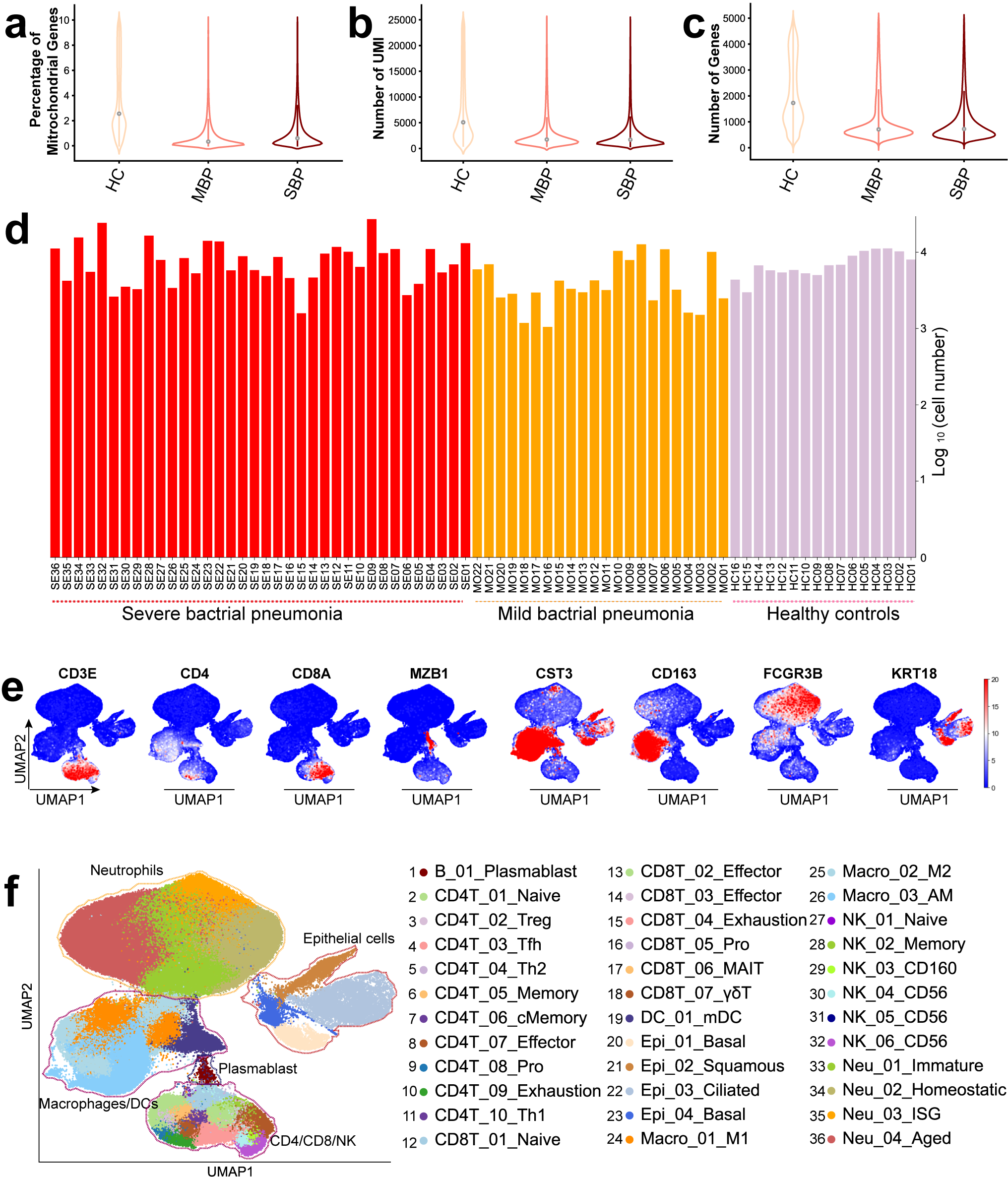
**

**Supplementary Fig 2. Detailed data output and visualization of single-cell transcriptional profiling of BALFs from 74 subjects, related to figure 1.**

a-c. Percentage of mitochondrial transcripts per cell (a), distribution of unique molecular identifier (UMI) counts per cell (b) gene counts per cell (c) detected for cells in each disease group.

d. Box plots illustrating the log_10_ transformed number of cells for each sample.

e. Canonical cell markers were used to label clusters by cell identity as represented in the UMAP plot. Colored according to normalized expression levels and legend labeled in log scale.

f. The clustering result (Left row) of 36 cell subtypes (right row) from 74 samples. Each point represents one single cell, colored according to cell type.


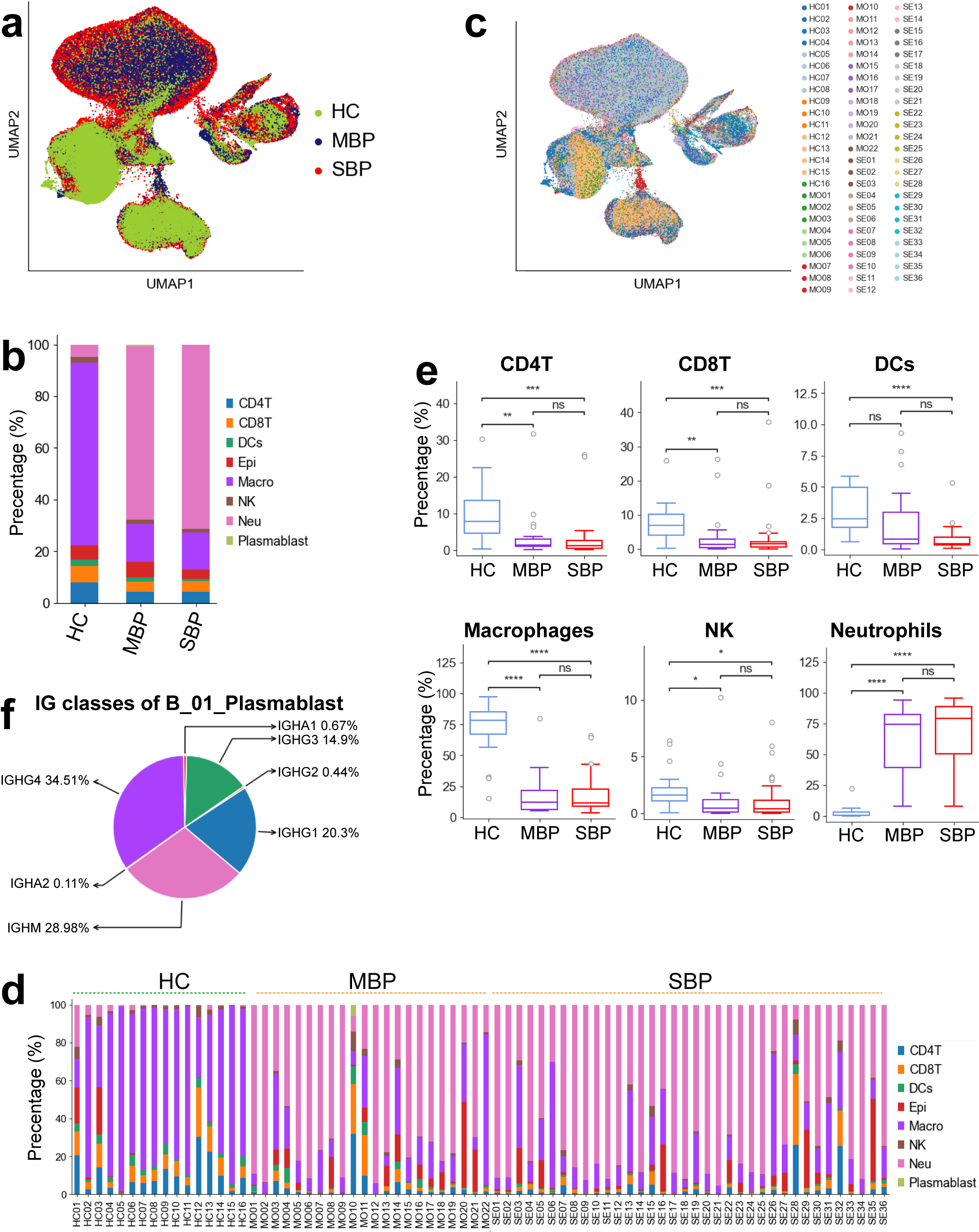


**Supplementary Fig 3. Quality of clustering and immune cell changes in BALFs for healthy donors and bacterial pneumonia patients, related to figure 1.**

a. UMAP of all cells colored by disease group.

b. Average proportion of each major cell type derived from HC (n=16), MBP (n=22) and SBP (n=36) samples.

c. UMAP of all cells colored by sample identity.

d. Bar plot showing the major cell compositions in each individual sample.

f. Classes of heavy chains for plasma cells.

e. Condition preference of each major cluster. y axis, average percentage of samples across three conditions. Conditions are shown in different colors. Significance was evaluated using the Kruskal-Wallis test with Bonferroni correction Significance was evaluated using the Kruskal-Wallis test with Bonferroni correction (*p<0.05, **p<0.01, ***p<0.001, ****p<0.0001, ^ns^p>0.05).


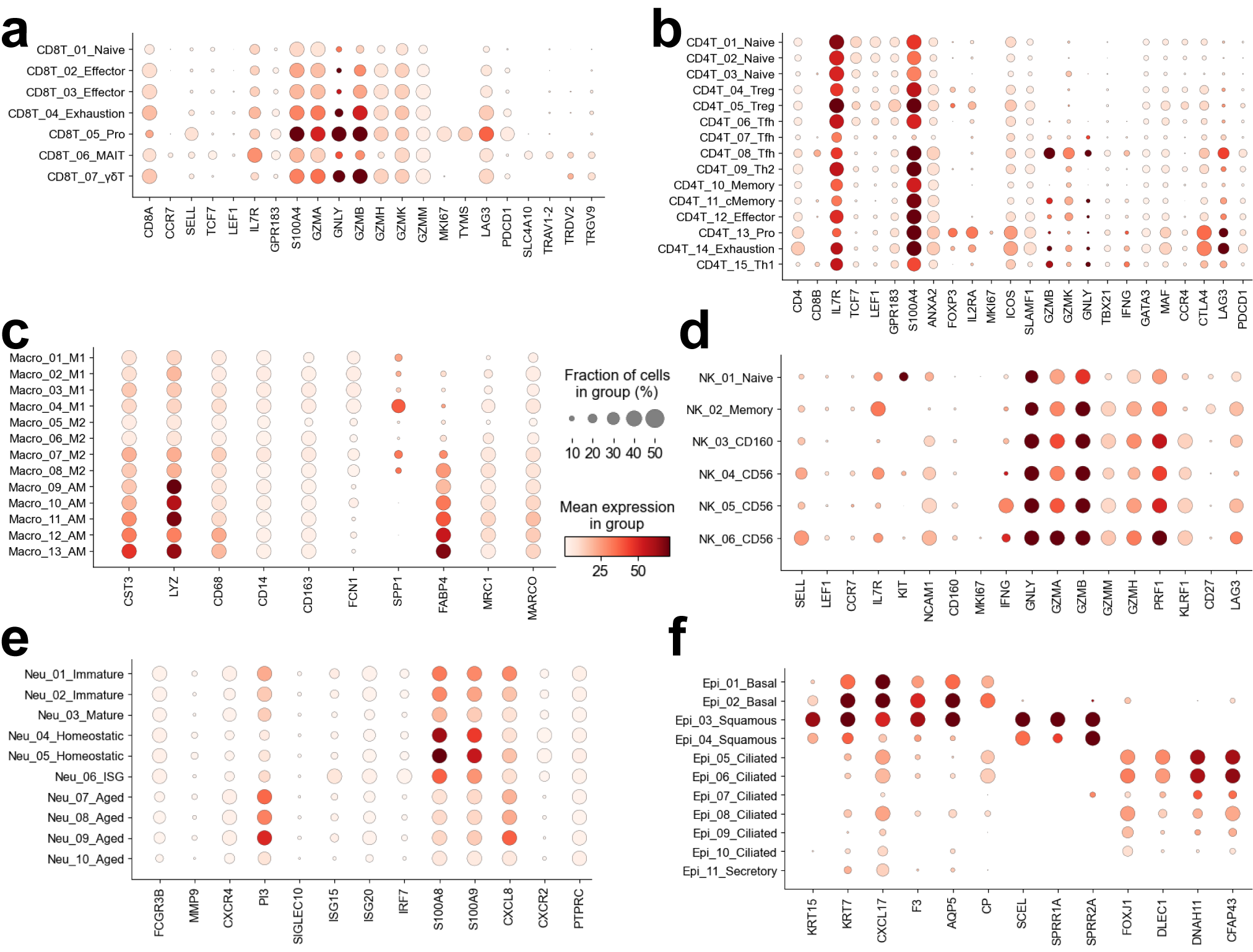


**Supplementary Fig 4. The selected markers of cell subsets in different major cell lineages, related to figure 1.**

a-f. Dot plots of selected marker genes (Rows) for cell subsets (Columns) within each cell lineage, including CD8^+^T (a), CD4^+^T (b), macrophages (c), NK cells (d), neutrophils (e) and epithelial (f) cell subsets.


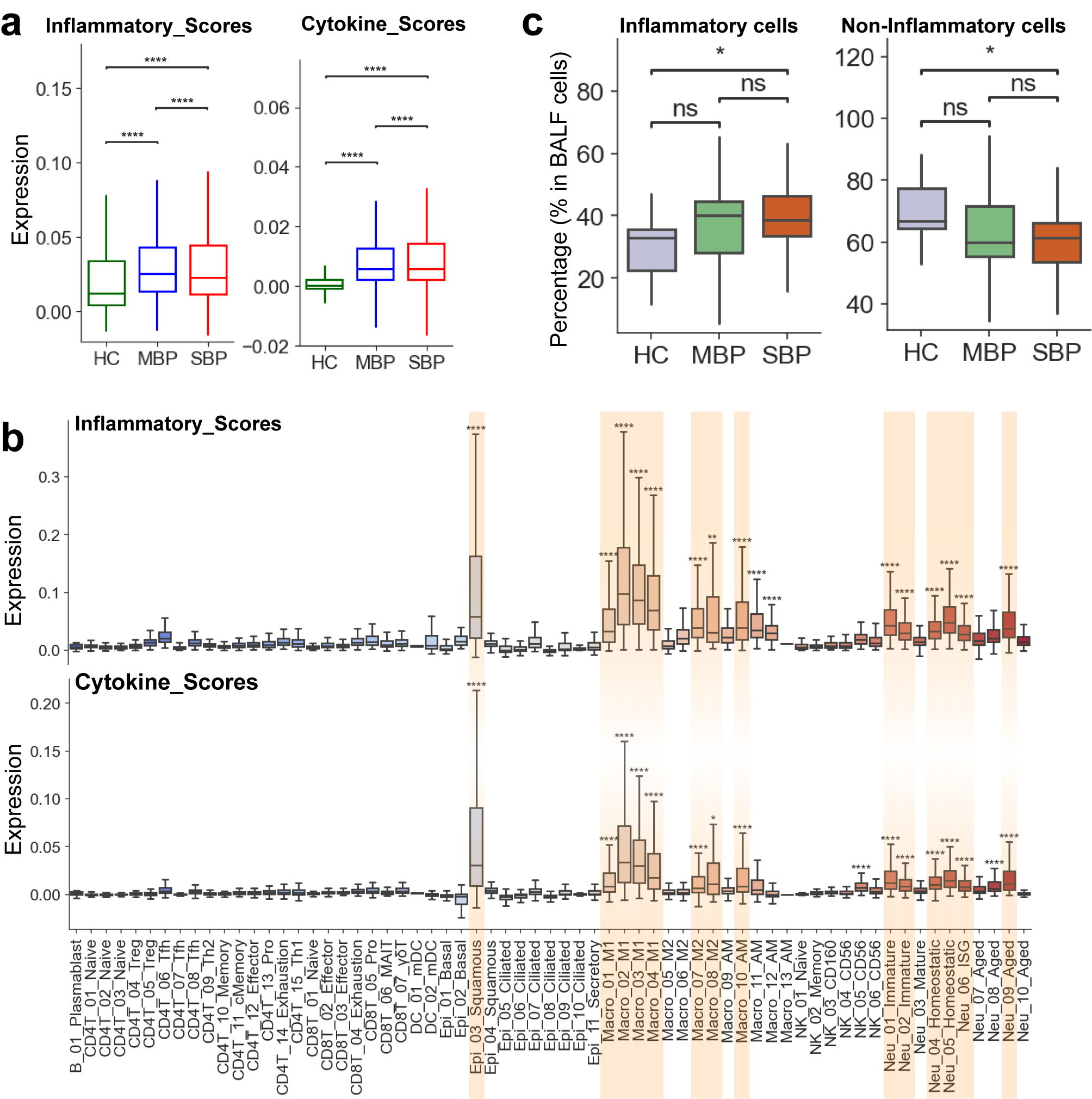


**Supplementary Fig 5. Identification of hyper-inflammatory subtypes associated with potential inflammatory response in BALFs, related to** **Figure 2**

a. Box plots showing the expression levels of inflammatory score (top panel) and cytokine score (bottom panel) derived from healthy donors, mild cases and severe cases. Horizontal lines represent median values, with whiskers extending to the farthest data point within a maximum of 1.5 × interquartile range. Significance was evaluated using the Kruskal-Wallis test with Bonferroni correction (*p<0.05, **p<0.01, ***p<0.001, ****p<0.0001, ^ns^p>0.05).

b. Boxplots showing the inflammatory score (top panel) and cytokine score (bottom panel) of cell subtypes. Significance was evaluated using the Kruskal-Wallis test with Bonferroni correction (*p<0.05, **p<0.01, ***p<0.001, ****p<0.0001, ^ns^p>0.05).

c. Box plots showing the proportion of inflammatory cell types (Left panel) and other non-inflammatory cell types (Right panel) across conditions. Significance was evaluated using the Kruskal-Wallis test with Bonferroni correction (*p<0.05, **p<0.01, ***p<0.001, ****p<0.0001, ^ns^p>0.05).

**
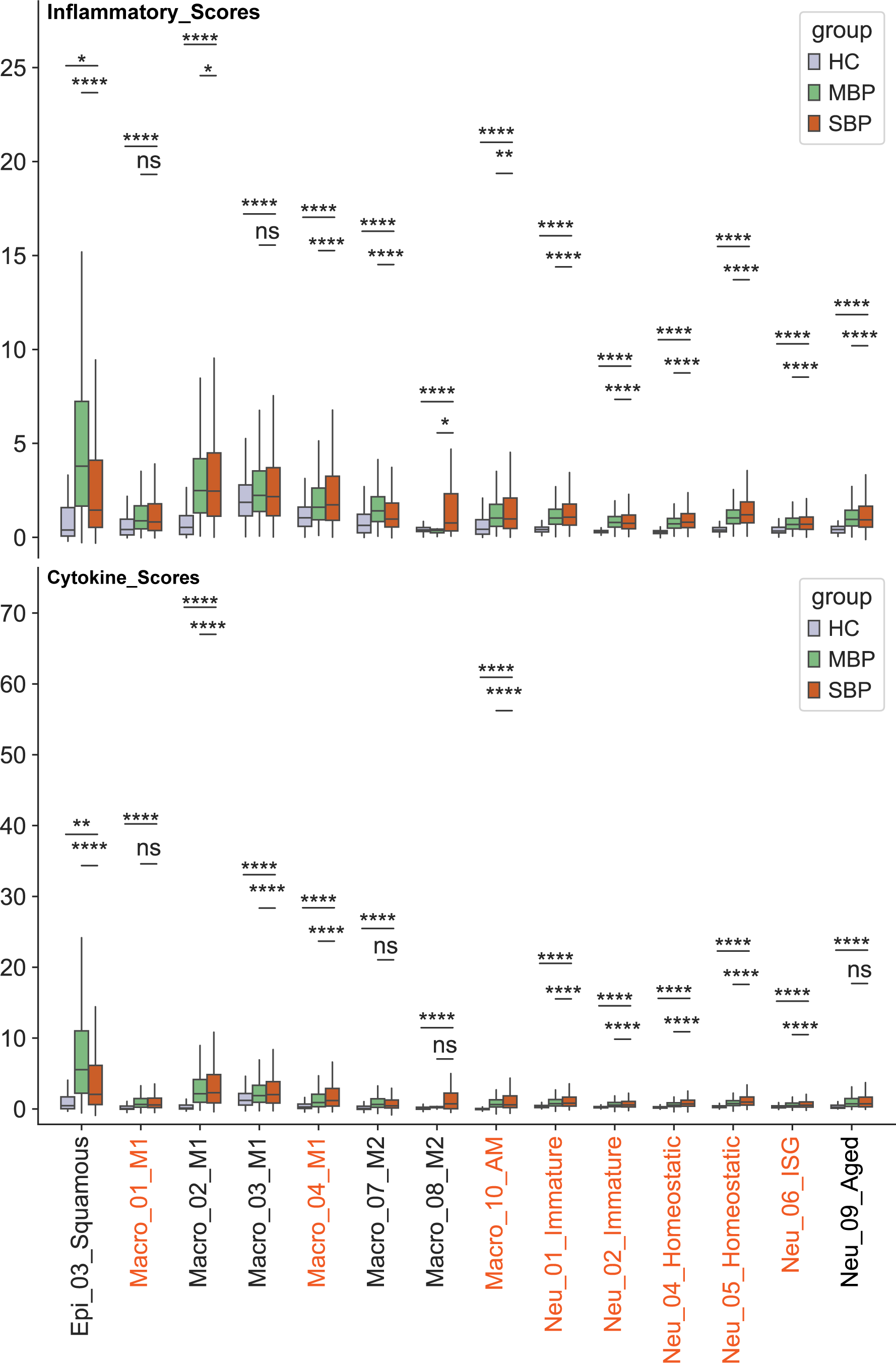
**

**Supplementary Fig 6. Identification of hyper-inflammatory subtypes associated with potential inflammatory response in BALFs, related to** **Figure 2**

Box plots showing the expression levels of inflammatory score (top panel) and cytokine score (bottom panel) derived from healthy donors, mild cases and severe cases. Horizontal lines represent median values, with whiskers extending to the farthest data point within a maximum of 1.5 × interquartile range. Significance was evaluated using the Kruskal-Wallis test with Bonferroni correction (*p<0.05, **p<0.01, ***p<0.001, ****p<0.0001, ^ns^p>0.05).


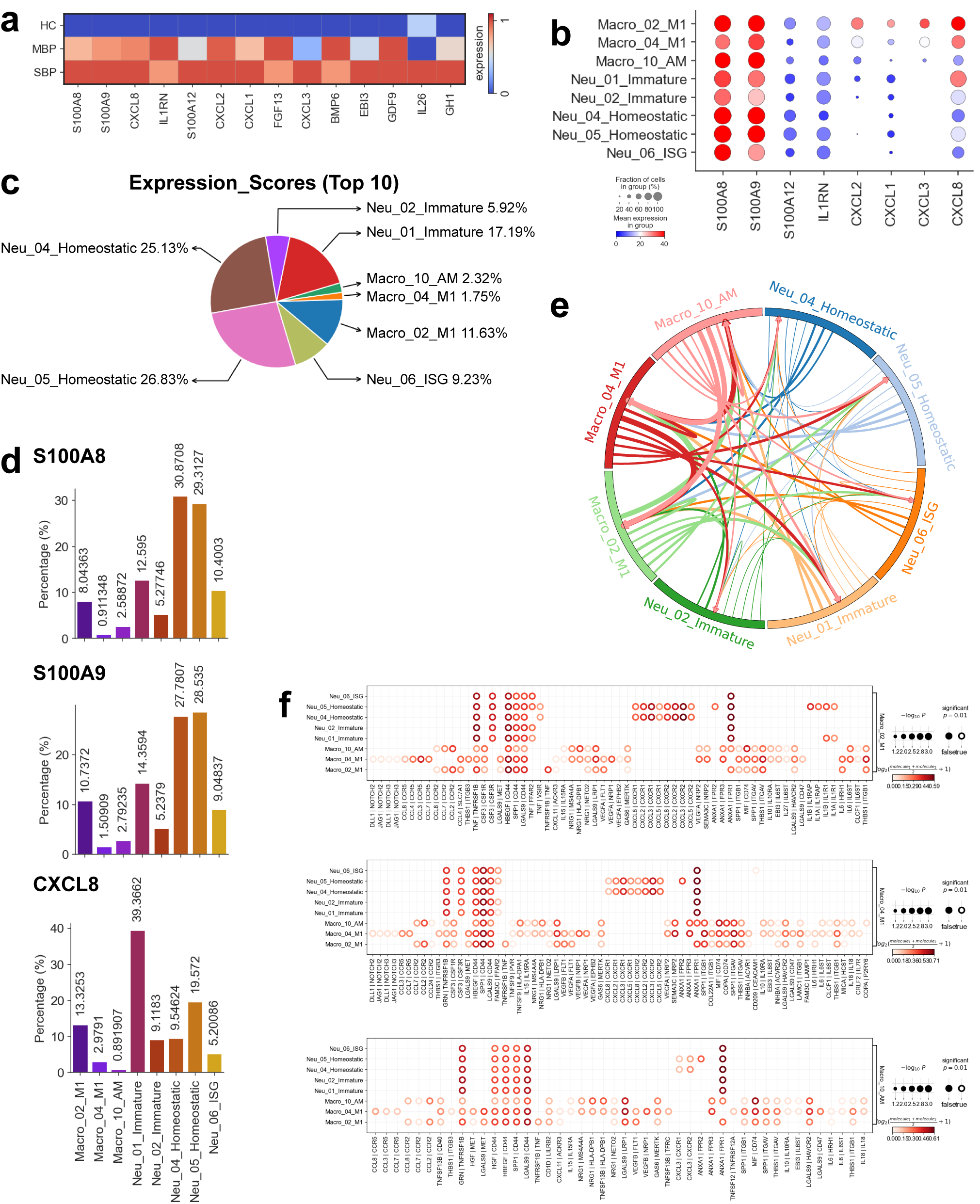


**Supplementary Fig 7. Details of hyper-inflammatory subtypes associated with potential inflammatory response in BALFs, related to Figure 2**

a. Heatmap depicting the expression of pro-inflammatory cytokines within hyper-inflammatory cell subtype identified across each disease condition.

b. Dot plots showing the selected genes (S100A8/A9/A12, IL1RN, CXCL2/1/8) in inflammatory cells.

c. Pie charts depicting the relative contribution of each inflammatory cell subtype to the top 10 cytokines.

d. Bar plots depicting the relative contribution of each inflammatory cell subtype to S100A8, S100A9 and CXCL8.

f. Dot plot depicting the ligand-receptor pair interactions between the 8 hyper-inflammatory cell subtypes and Macro_02_M1 (Top panel)/Macro_04_M1 (Middle panel)/Macro_10_AM (Bottom panel).

e. Circos plot depicting the ligand-receptor pair interactions between the 8 hyper-inflammatory cell subtypes.


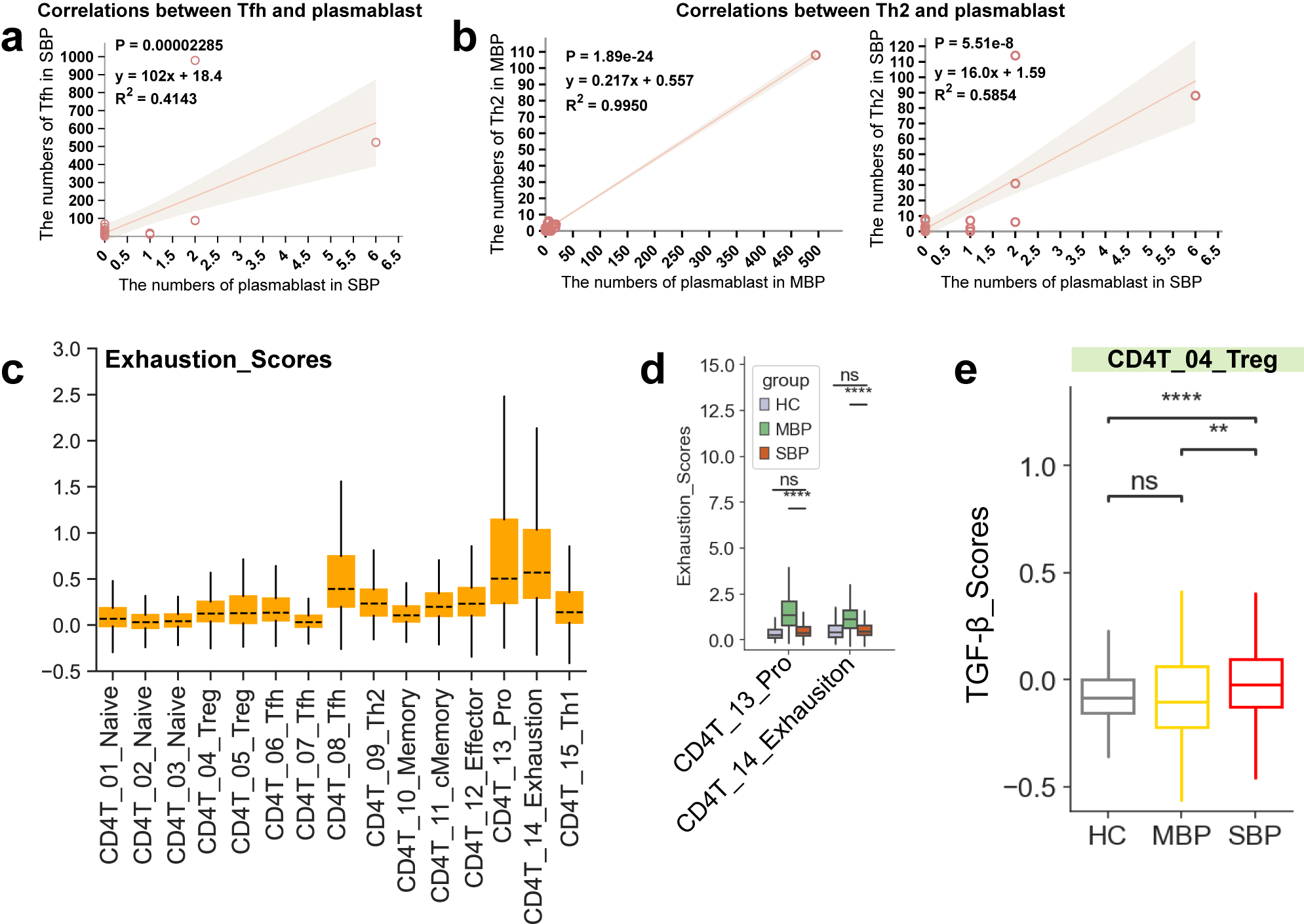


**Supplementary Fig 8. Characterization of gene expression differences in CD4^+^T cells across conditions, related to Figure 3**

a. Correlations between the compositions of plasmablast with Tfh cells in severe patients.

b. Correlations between the compositions of plasmablast with Th2 cells in mild (Left) and severe (Right) patients.

c. Box plots showing the indicated exhaustion score of CD4^+^T cell subtypes.

d. Boxplots showing the exhaustion score of CD4T_13_Pro and CD4T_14_Exhaustion across disease conditions. Significance was evaluated using the Kruskal-Wallis test with Bonferroni correction (*p<0.05, **p<0.01, ***p<0.001, ****p<0.0001, ^ns^p>0.05).

e. Boxplots showing the TGF-β score of CD4T_4_Treg across disease conditions. Significance was evaluated using the Kruskal-Wallis test with Bonferroni correction (*p<0.05, **p<0.01, ***p<0.001, ****p<0.0001, ^ns^p>0.05).


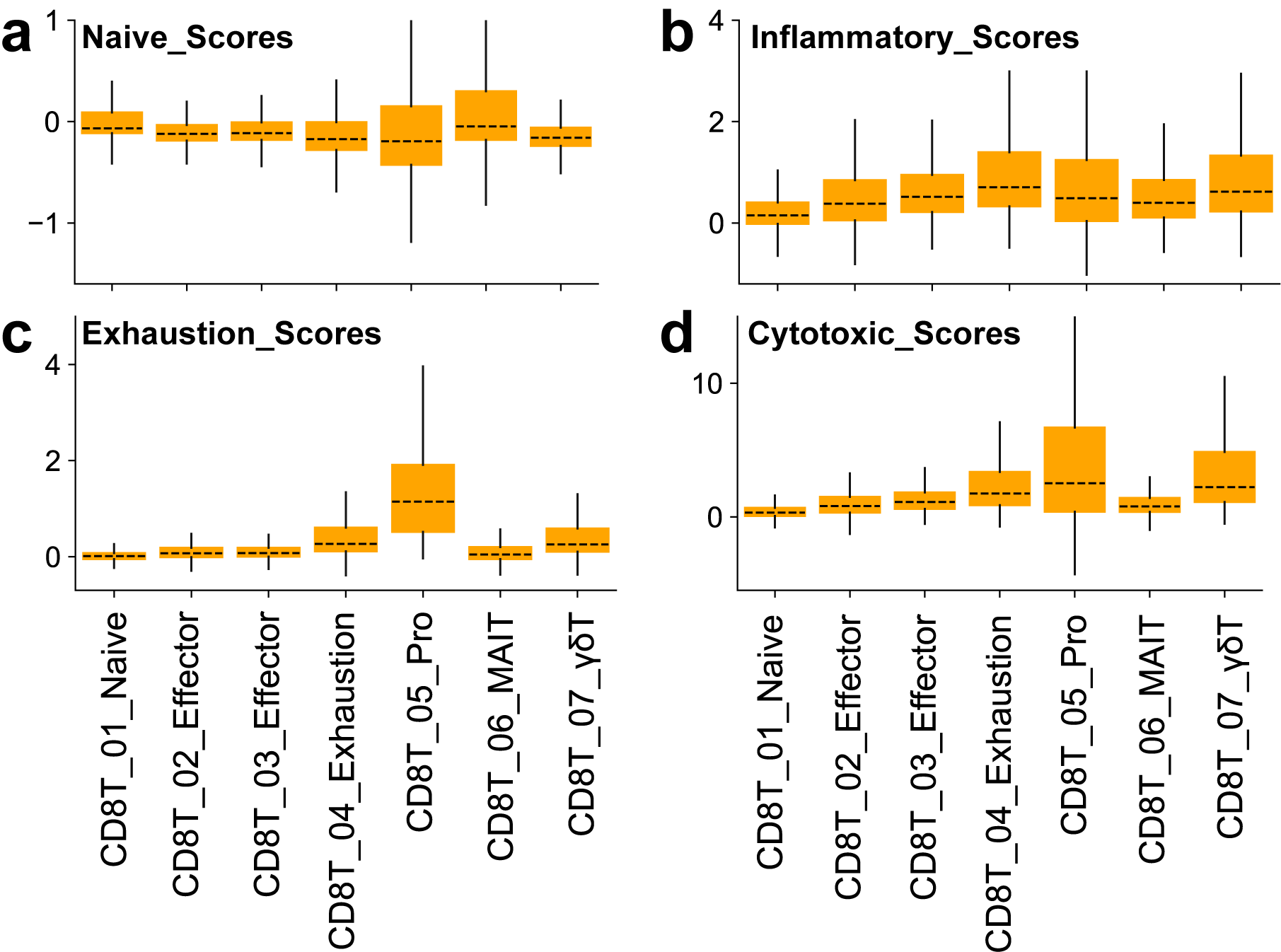


**Supplementary Fig 9. Characterization of gene expression differences in CD8^+^T cells across conditions, related to Figure 4**

a. Box plot showing the indicated naïve score of CD8^+^T cell subtypes.

b. Box plot showing the indicated inflammatory score of CD8^+^T cell subtypes.

c. Box plot showing the indicated exhausted score of CD8^+^T cell subtypes.

d. Box plot showing the indicated cytotoxic score of CD8^+^T cell subtypes.


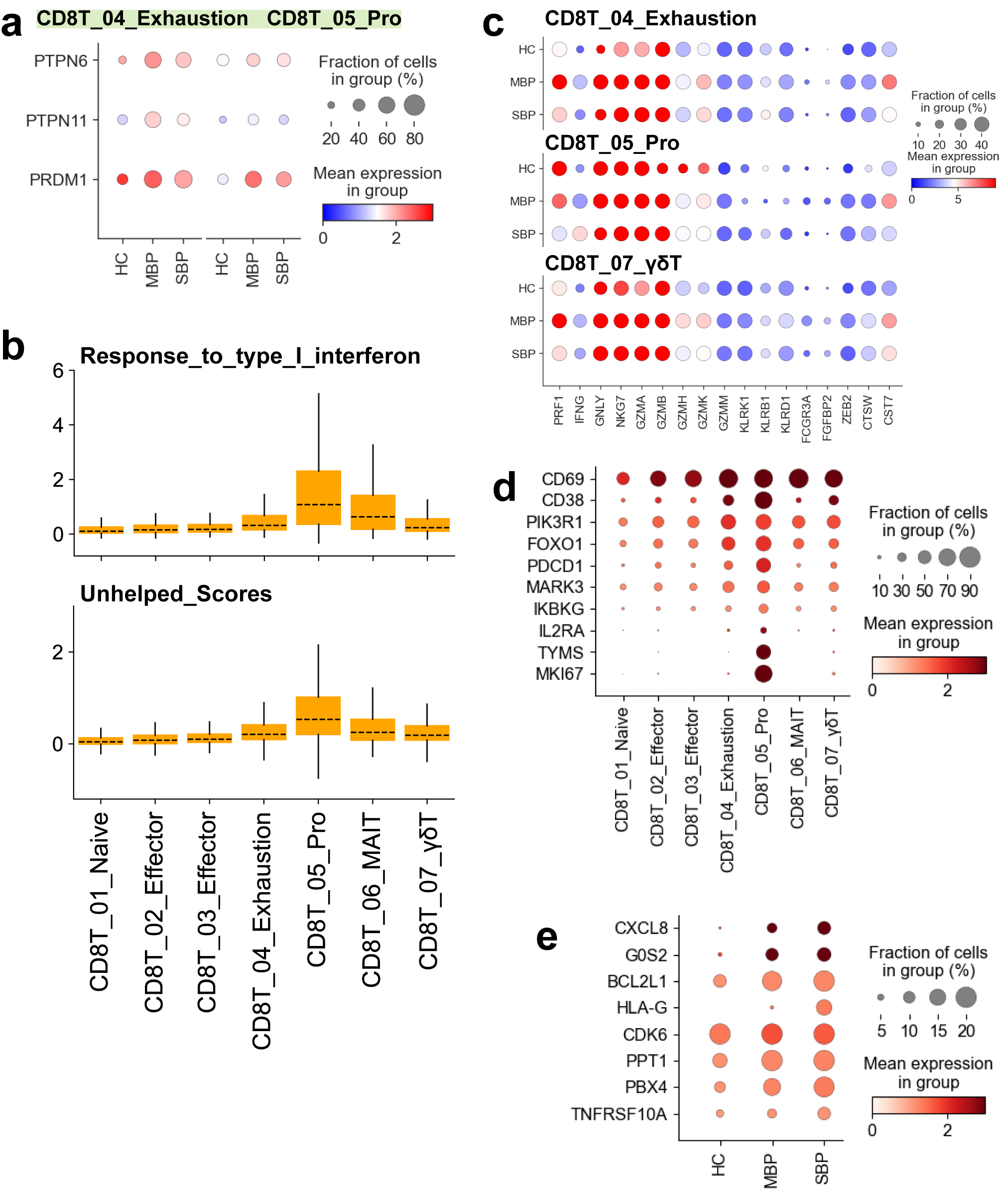


**Supplementary Fig 10. Characterization of gene expression differences in CD8^+^T cells across conditions, related to Figure 4**

a. Dot plots showing the expression of selected genes in CD8_04_Exhaustion and CD8_05 _Pro across disease conditions.

b. Box plot showing the indicated response to type I interferon (Top row) and unhelped (Bottom row) scores of CD8+T cell subtypes.

c. Dot plots showing the expression of selected genes in CD8_04_Exhaustion, CD8_05 _Pro and CD8T_07_γδT across disease conditions.

d. Dot plots showing the expression of selected genes in CD8^+^T cell subsets.

e. Dot plots showing the expression of selected genes in CD8^+^T across disease conditions.


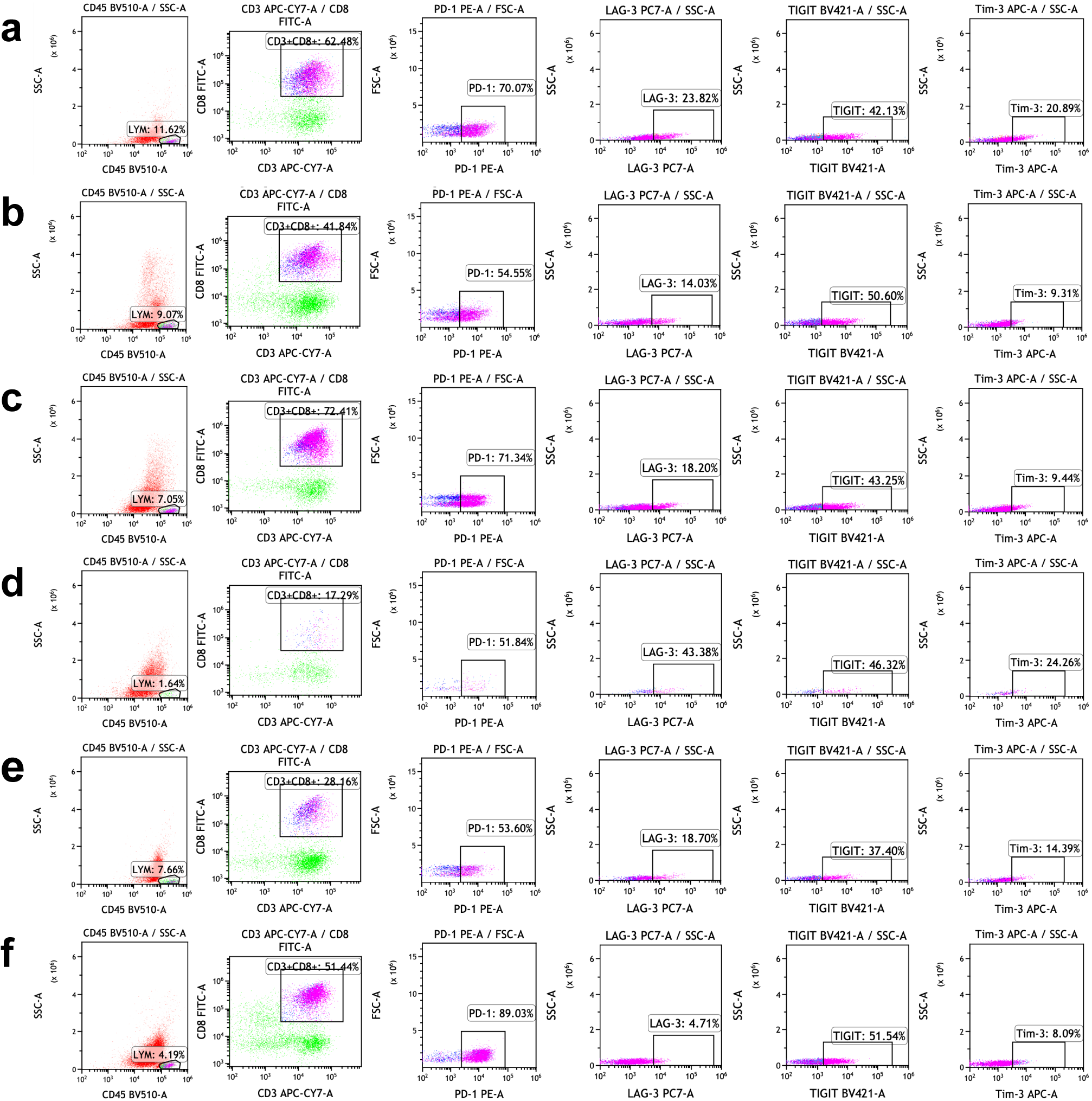


**Supplementary Fig 11. Flow cytometry plots showing gating strategy in CD8^+^T cells from patients with mild bacterial pneumonia, related to Figure 4.**

a-f. Flow cytometry plots showing gating strategy and typical exhausted molecules in CD8^+^T cells from 6 patients with mild bacterial pneumonia.

**
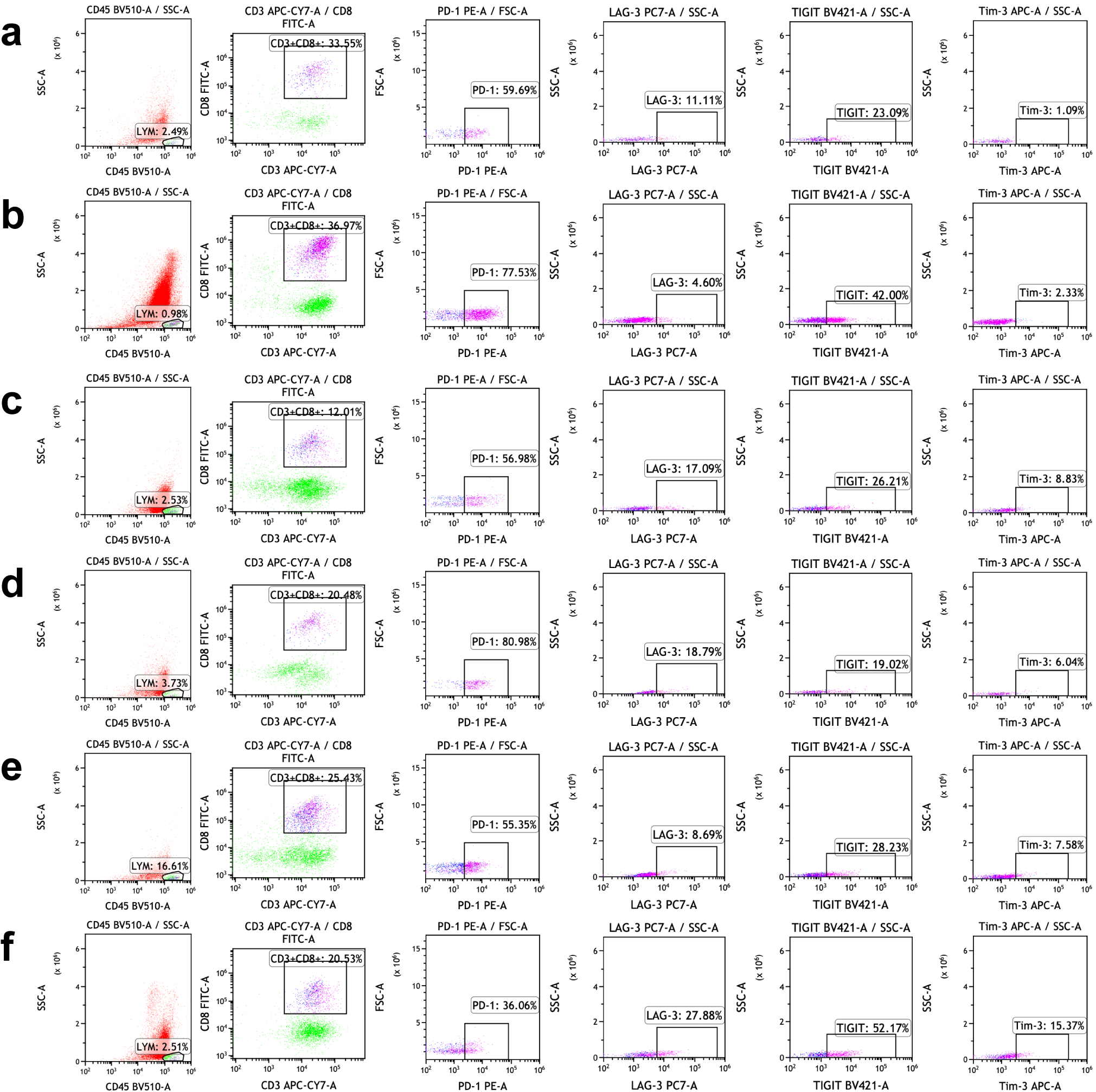
**

**Supplementary Fig 12. Flow cytometry plots showing gating strategy in CD8^+^T cells from patients with severe bacterial pneumonia, related to Figure 4.**

a-f. Flow cytometry plots showing gating strategy and typical exhausted molecules in CD8^+^T cells from 6 patients with severe bacterial pneumonia.

**
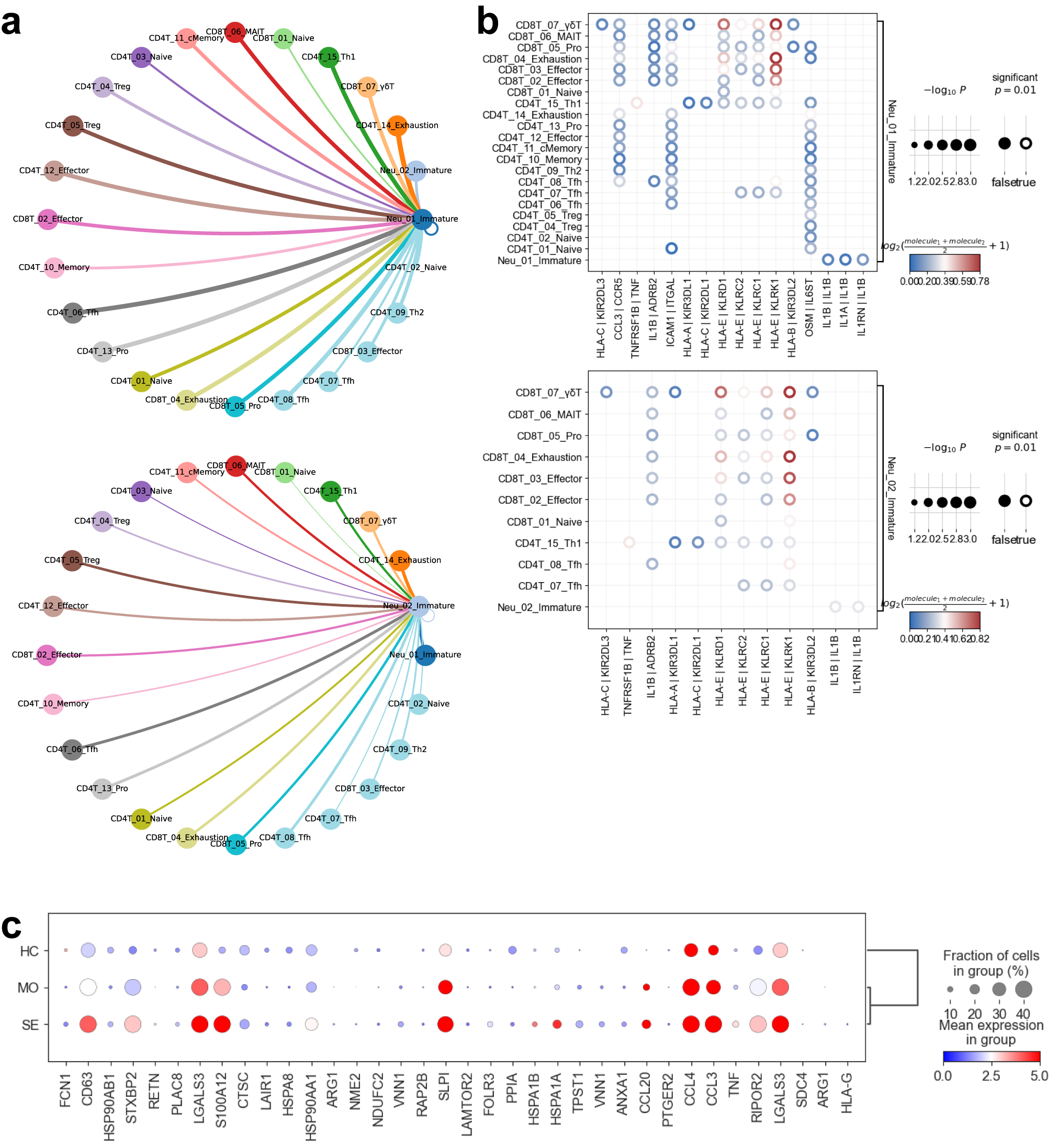
**

**Supplementary Fig 13. Characterization of gene expression differences in neutrophils across conditions, related to Figure 5**

a. Shell plots showing the interaction between Neu_01_Immature (Top) and T cell subsets, as well as Neu_02_immature (Bottom) and T cell subsets in severe patients.

b. Dot plot showing the interactions between Neu_01_Immature (Top) and T cell subsets, as well as Neu_02_immature (Bottom) and T cell subsets in severe patients.

c. Dot plot of selected genes in neutrophils across disease conditions.


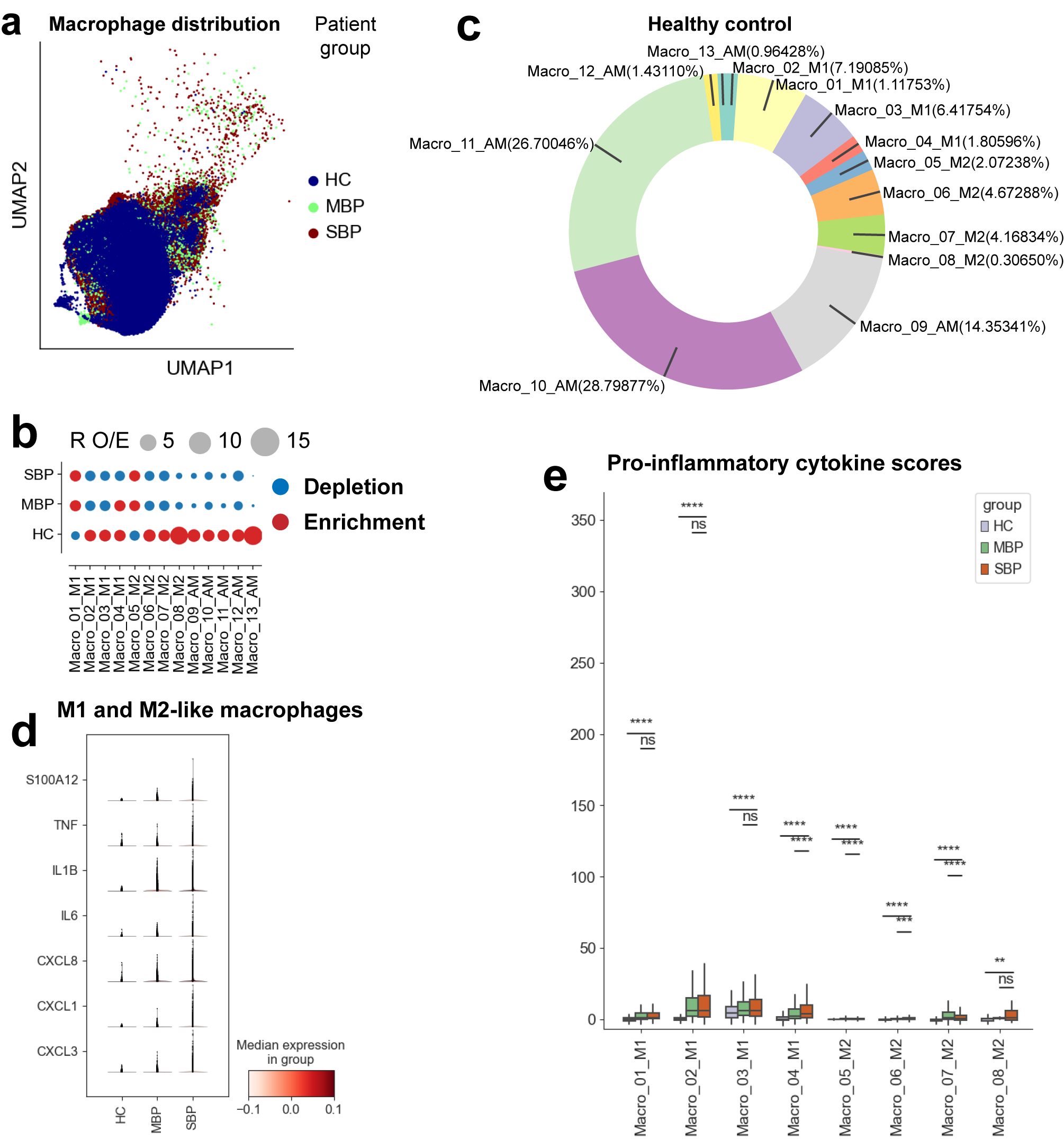


**Supplementary Fig 14. Characterization of gene expression differences in macrophages across conditions, related to Figure 6**

a. UMAP plots of macrophage colored based on disease distribution.

b. Disease preference of macrophage clusters as estimated using R_O/E_.

c. Pie charts depicting the relative percentage of each macrophage subtype in healthy controls.

d. Violin plots of selected genes in M1- and M2-like macrophages across disease condition.

e. Bar plots of pro-inflammatory scores in M1- and M2-like macrophage subsets across disease condition. Significance was evaluated using the Kruskal-Wallis test with Bonferroni correction (*p<0.05, **p<0.01, ***p<0.001, ****p<0.0001, ^ns^p>0.05).

**
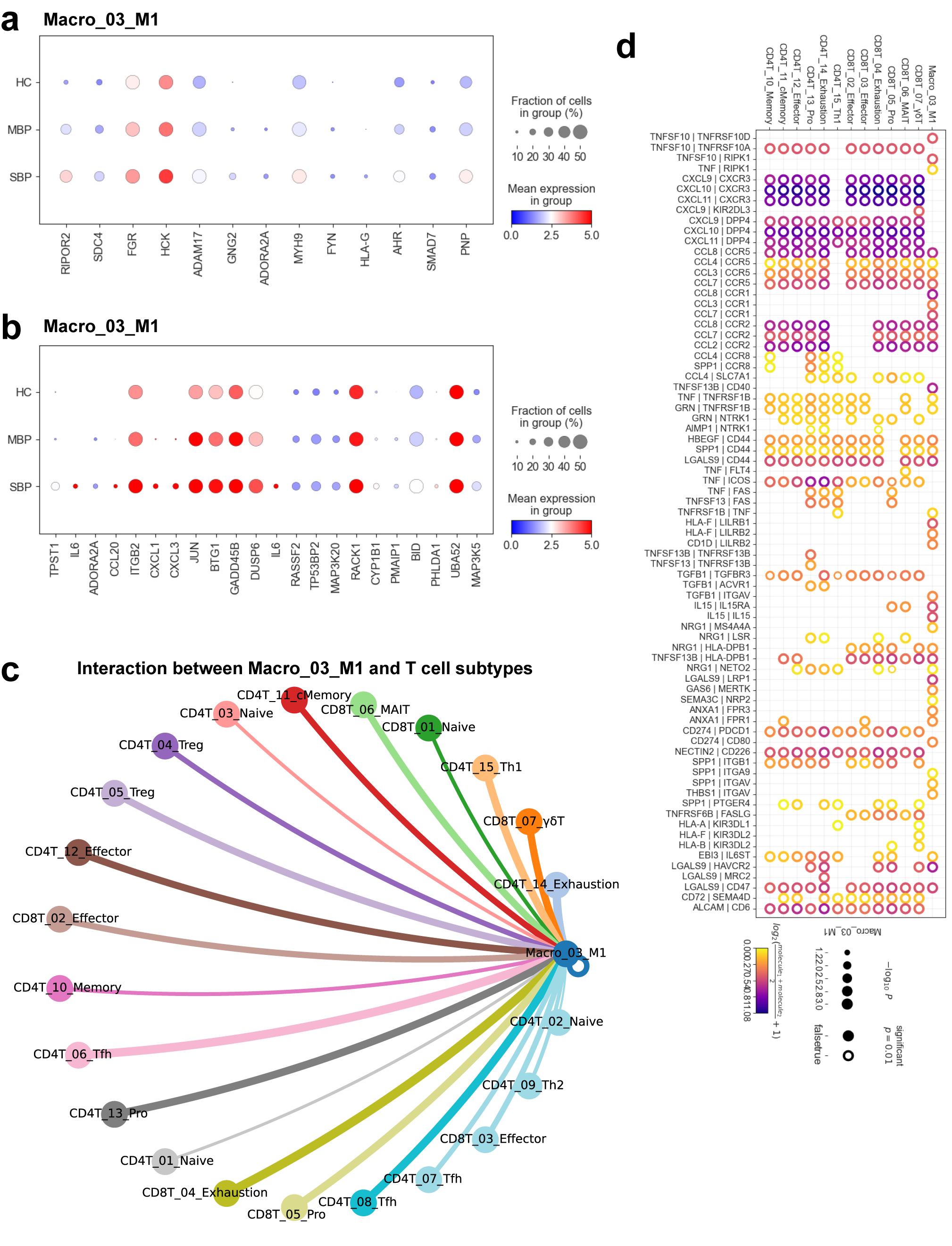
**

**Supplementary Fig 15. Characterization of gene expression differences in macrophages across conditions, related to Figure 6**

a. Dot plot showing selected genes with immunosuppressive functions in the Macro_03_M1 subset.

b. Dot plot showing selected inflammatory-related genes in the Macro_03_M1 subset.

c. Shell plots showing the interaction between Macro_03_M1 subset and T cell subsets in severe patients.

d. Dot plot of the interactions between Macro_03_M1 subset and T cell subsets in severe patients.


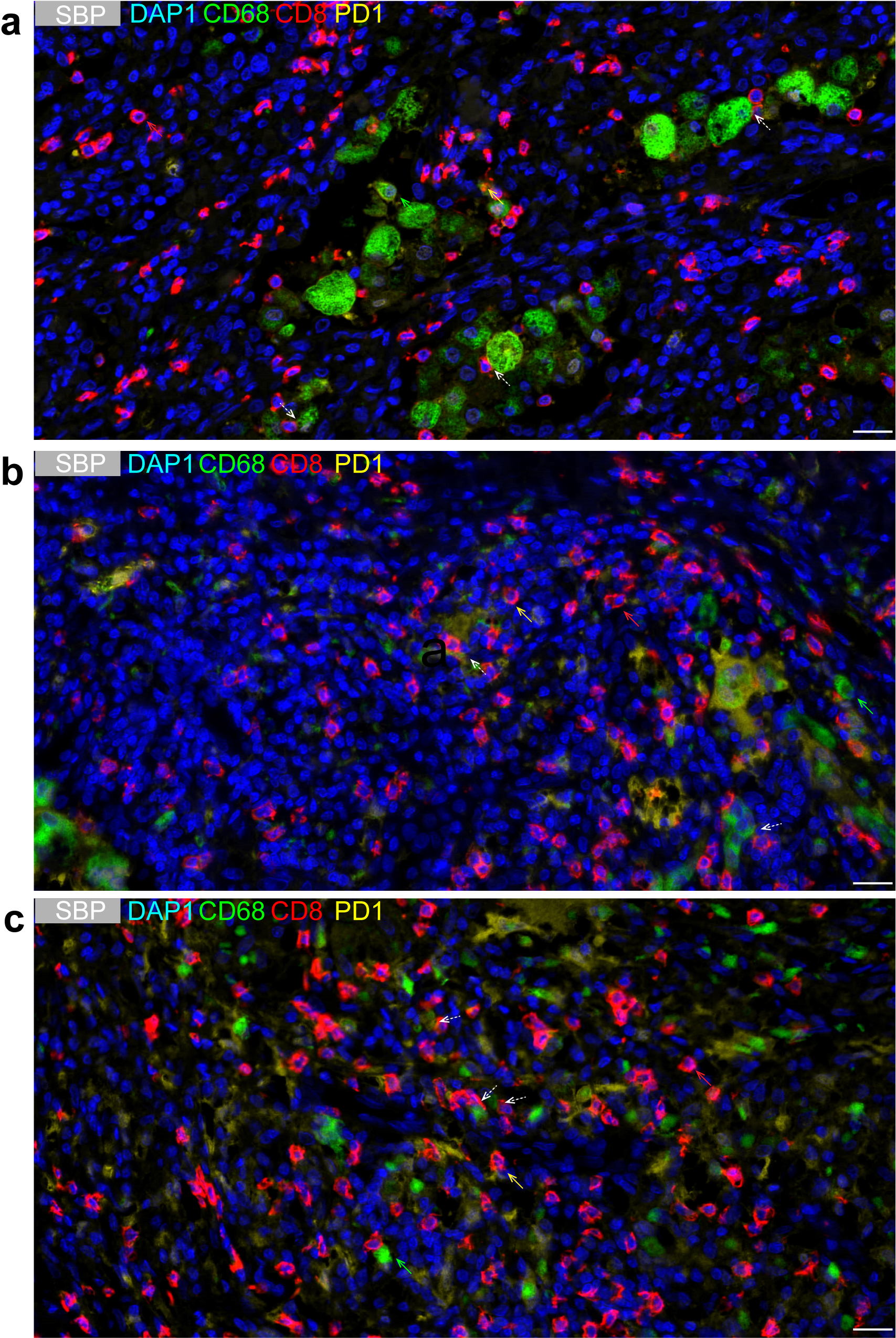


**Supplementary Fig 16. Multicolour IHC staining of Macrophages and exhausted CD8^+^ T-cells in the lung, related to Figure 6.**

a-c. Three representative multicolour immunohistochemical (IHC) stained lung section from the SBP group are shown. Macrophages, CD8^+^ T-cells, and CD8^+^PD1^+^ T-cells are indicated by green, red, and yellow solid arrows, respectively. Interactions between macrophages and CD8^+^ T cell subsets are highlighted with white dotted arrows. Scale bars, 20 μm.
